# A synthetic peptide library for benchmarking crosslinking-mass spectrometry search engines for proteins and protein complexes

**DOI:** 10.1038/s41467-020-14608-2

**Published:** 2020-02-06

**Authors:** Rebecca Beveridge, Johannes Stadlmann, Josef M. Penninger, Karl Mechtler

**Affiliations:** 1 Research Institute of Molecular Pathology (IMP), Vienna Biocenter (VBC), Campus-Vienna-Biocenter 1, 1030 Vienna, Austria; 2 Institute of Molecular Biotechnology of the Austrian Academy of Sciences (IMBA), Vienna Biocenter (VBC), Dr. Bohr-Gasse 3, 1030 Vienna, Austria; 3 Department of medical Genetics, Life Sciences Institute, University of British Columbia, Vancouver Campus, 2350 Health Sciences Mall, Vancouver, BCV6T 1Z3 Canada

**Keywords:** Proteomics, Protein-protein interaction networks, Mass spectrometry, Chemical libraries

## Abstract

Crosslinking-mass spectrometry (XL-MS) serves to identify interaction sites between proteins. Numerous search engines for crosslink identification exist, but lack of ground truth samples containing known crosslinks has precluded their systematic validation. Here we report on XL-MS data arising from measuring synthetic peptide libraries that provide the unique benefit of knowing which identified crosslinks are true and which are false. The data are analysed with the most frequently used search engines and the results filtered to an estimated false discovery rate of 5%. We find that the actual false crosslink identification rates range from 2.4 to 32%, depending on the analysis strategy employed. Furthermore, the use of MS-cleavable crosslinkers does not reduce the false discovery rate compared to non-cleavable crosslinkers. We anticipate that the datasets acquired during this research will further drive optimisation and development of XL-MS search engines, thereby advancing our understanding of vital biological interactions.

## Abstract (web summary)

Validating crosslinking-mass spectrometry workflows is hampered by the lack of a ground truth to assess the robustness of the crosslink identifications. Here, the authors present a synthetic library of crosslinked peptides, enabling unambiguous discrimination of correct and incorrect crosslink identifications.

## Introduction

Chemical crosslinking combined with mass spectrometry (XL-MS) is frequently used to gain structural information on proteins and protein complexes^1,2^. In a crosslinking experiment, a reagent forms covalent bonds between specific amino acid side-chains that are in close spatial proximity, thus revealing distance restraints between residues, and hence interaction sites within a protein or between different proteins^3,4^. Initial applications of these techniques were limited to small proteins and protein complexes. More recently, due to major methodological and technological developments, XL-MS has been applied to living cells to investigate protein interactions and topological structures at the proteome-wide level^5–8^.

For studies on small proteins and protein complexes, standard crosslinking reagents disuccinimidyl suberate (DSS), bis-(sulfosuccinimidyl) suberate (BS3), or bis-(sulfosuccinimidyl) glutarate (BS2G) are typically used which react with lysine residues and N-termini of proteins, and to a lesser extent with serine, threonine, and tyrosine. Several complications arise during the identification of crosslinked peptides with such reagents. Measurement of the intact mass of the crosslinked moiety, rather than the mass of the individual peptides, quadratically increases the number of possible peptide pairings to be searched with the number of peptides in the database (often referred to as the ‘*n*-squared’ problem), and is thought to reduce confidence in the assignment of crosslinked peptides^9^. With the aim of overcoming this limitation, MS-cleavable crosslinkers such as disuccinimidyl dibutyric urea (DSBU) and disuccinimidyl sulfoxide (DSSO) were introduced^10,11^. This class of reagents contains MS-labile bonds at either side of a functional group within their spacer regions that can be selectively and preferentially fragmented prior to peptide backbone cleavage during collision induced dissociation (CID) or higher-collision induced dissociation (HCD). Gas-phase cleavage of the crosslinking reagent during tandem MS enables MS3 acquisition methods, which facilitate peptide sequencing using traditional database search engines due to circumvention of the *n*-squared problem. Additionally, cleavable crosslinkers generate diagnostic ion doublets during MS2, which are required for the use of novel database search engines such as XlinkX and MeroX that provide means for proteome-wide XL-MS studies^12,13^.

Another critical challenge during XL-MS lies in estimating the error rate in a search for crosslinked peptides. In protein identification by mass spectrometry an FDR (false discovery rate) method is often used, whereby incorrect decoy sequences added to the search space correspond with incorrect search results which might otherwise be deemed correct. This allows the estimation of how many incorrect results are in a final dataset. In XL-MS, the FDR estimation is complicated by the fact that every match is a combination of two peptides, each with its own probability to be false. Further, false discovery rates can be estimated at different points during data analysis, for example, protein or residue pairs, and misuse of these approaches can lead to a higher error in the results than is targeted in the search^9^.

Owing to the versatility of XL-MS techniques, many algorithms have been developed to identify crosslinks from mass spectrometry datasets. For example, algorithms used in conjunction with non-cleavable, non-labelled crosslinkers include (but are not limited to) pLink^14^, StavroX^15^, Xi^16^ and Kojak^17^. For MS-cleavable reagents, XlinkX^12^ and MeroX^13^ are often used. pLink, StavroX, Xi, MeroX and XlinkX have an inbuilt FDR calculator, whereas Kojak relies on external tools to estimate FDRs such as Percolator^18^ or PeptideProphet^19^ which is incorporated into the trans proteomic pipeline^20^.

Crosslinks are often validated by mapping them onto crystallographic models and measuring the distance between the α-carbon atoms of the crosslinked residues^10,11,21,22^. Distances that are within a defined cut-off point are considered to be true, and those that exceed this distance limit, and are therefore in disagreement with the structural model, are regarded as false positives. One limitation of such methods to test bioinformatic approaches is that the formation of non-specific crosslinks during the experimental procedure cannot be fully eliminated, and such a crosslink would wrongly be defined as a false positive. Furthermore, such an approach often under-estimates FDR since the crosslinks that are evaluated are limited to those that are formed between peptides contained in the same protein model^23^. This lack of a generally accepted method on how to control for falsely identified crosslinks may have severe implications that are far-reaching in biological research.

Several elegant benchmarking approaches were utilised by Chen et al.^24^ during the evaluation of pLink 2. Simulated datasets, synthetic datasets^25^, ^15^N metabolically labelled datasets and entrapment datasets^17,26^ were used to compare the precision and sensitivity of pLink 2 compared to other search engines. The synthetic dataset was obtained by taking 38 synthetic peptides derived from the sequence of the UTP-B protein and crosslinking them in one reaction. Data were searched against increasingly larger databases, and the sensitivity and precision of pLink 1, pLink 2 and Kojak were reported.

To date, comprehensive assessment of the accuracy and sensitivity of XL-search algorithms have been hindered by a lack of ground truth data that can be used to determine whether crosslinks were correctly or incorrectly assigned. To overcome this problem, we have constructed a synthetic library of up to 426 crosslinked peptides. This allows the unambiguous discrimination of XL-MS spectra that are correctly, incorrectly, and not identified across all search engines developed for the identification of crosslinks from the MS data. In the following scenarios, results are filtered to an estimated FDR of 5% at the CSM level, and the actual FDR is subsequently calculated based on the design of the library. For search engines with inbuilt validation strategies for error estimation (pLink, Xi and StavroX), calculated false crosslink identification ranges from 5.2% to 11.3%. When different validation strategies (Percolator and PeptideProphet) are applied to the same Kojak search output, calculated false crosslink identification ranges from 2.4% to 32%. False crosslink identification by MeroX is calculated to be 4.9% or 8.3% for Rise and Riseup mode, respectively. When recommended score cut-off values are used for XlinkX, calculated false identification ranges from 0% (with stepped-HCD MS acquisition strategy) to 6.2% (employing MS2-MS3).

## Results

### Design of the crosslinked peptide library

**Fig. 1:**
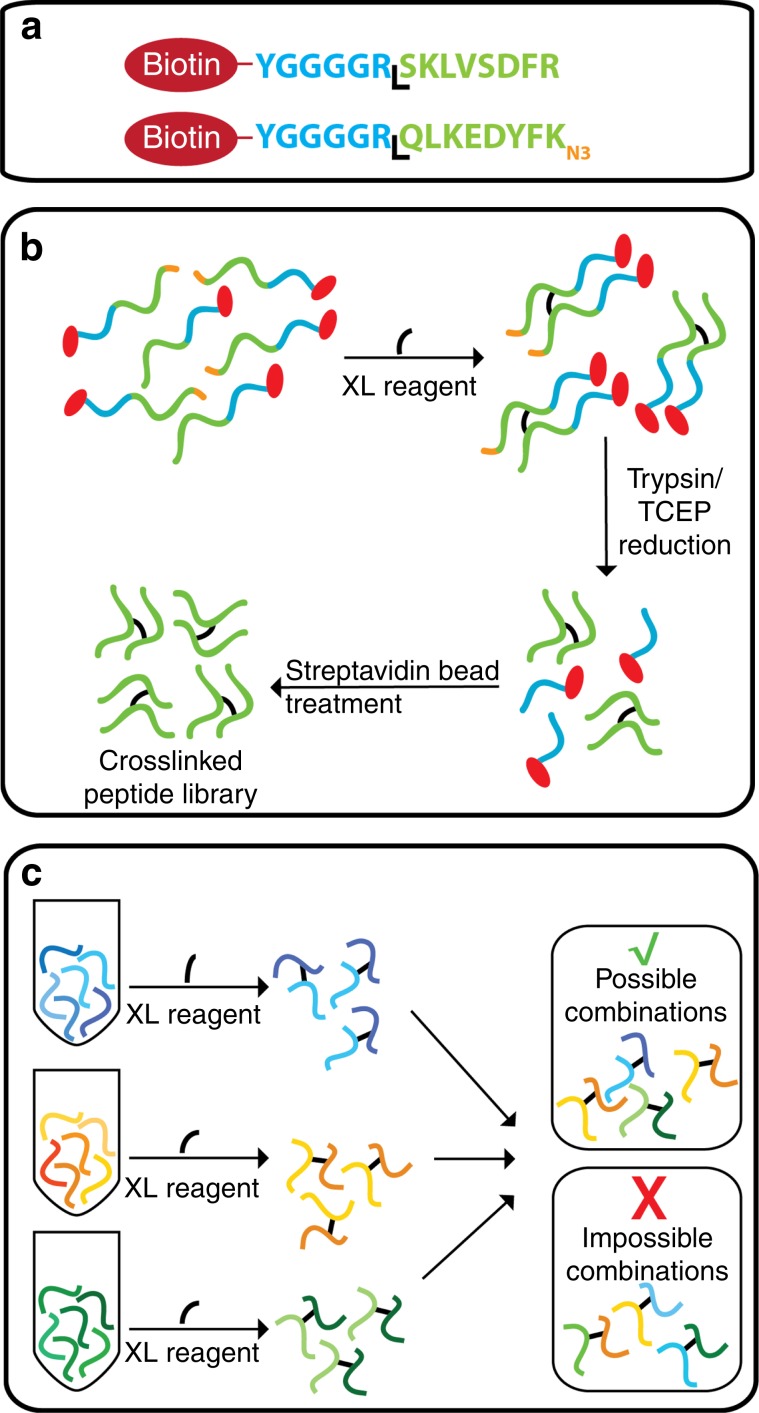
Design of the crosslinked peptide library. **a** the N-terminal amine group is protected from crosslinking with a biotin group that is followed by a linker region with a tryptic cleavage site. C-terminal lysine residues are incorporated with an azide group to prevent crosslinking to this site. **b** Peptides are crosslinked, treated with trypsin and the azide groups on the C-terminal lysine residues are reduced to an amine. Biotin-linker groups are removed from the solution with streptavidin beads. **c** The synthetic peptides are crosslinked in separate groups and combined prior to LC-MS analysis.

The crosslinked peptide library was based on the amino acid sequences of tryptic peptides from *S. pyogenes* Cas9 and was chemically synthesised according to Fig. 1. Ninety-five peptides were selected for synthesis that were 5–20 residues long, containing a single lysine residue for crosslinking purposes. To prevent peptide N-termini and C-terminal lysine residues from crosslinking during formation of the library, any C-terminal lysine residues were incorporated into the peptide as epsilon-azido-l-lysine, and the N-terminus of the peptide was protected from crosslinking with a biotin group covalently linked to a generic ‘linker’ peptide sequence YGGGGR, followed by the library peptide sequence (Fig. 1a). Subsequently, the only crosslinker-reactive site in each peptide at the time of crosslinking was one single lysine residue. Peptides were crosslinked with the gradual addition of crosslinking reagent to favour the formation of crosslinks (as opposed to monolinks that would likely form if all the XL reagent was added at once). The crosslinked peptides were then treated with trypsin overnight to enzymatically cleave the N-terminal biotin-linker peptide region, and subsequently treated with TCEP to reduce the C-terminal lysine azido-group to an amine. After trypsin digestion, the most abundant species in the solution was the biotin-linker, which strongly interfered with MS measurements, limiting the amount of material that could be loaded onto the analytical column. The biotinylated peptide species was therefore removed with streptavidin beads (Fig. 1b), and the resulting mixture exclusively comprised of crosslinked and monolinked tryptic peptides. Incompletely processed peptides, as well as peptides crosslinked via serine, threonine and tyrosine residues, did not feature highly in our results and were therefore not considered in our analysis.

To create a challenging dataset for the assessment of XL-search algorithms, the synthetic peptides were divided into 12 groups for crosslinking, which were then combined prior to analysis via LC-MS. This theoretically gives rise to 426 potential crosslinks. It can be inferred that crosslinks identified between two peptides of the same group can be correct, while those identified between two peptides from separate groups are known to be false positives (Fig. 1c).

### Assessment of search engines for non-cleavable crosslinkers

**Fig. 2:**
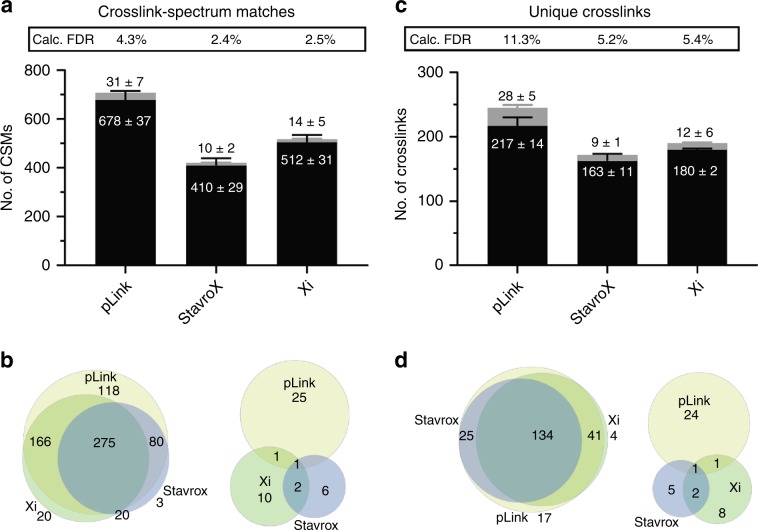
Comparison of pLink, StavroX and Xi performance based on the crosslinked peptide library. **a** number of CSMs that correspond to correct (black) and incorrect (grey) crosslinks identified by three search algorithms with incorporated FDR estimation. Results were filtered to an estimated 5% CSM-FDR, and the calculated FDR is given for each algorithm. **b** Agreement of correct (left) and incorrect CSMs (right) between pLink, StavroX and Xi for one technical repeat. **c** Number of correct unique crosslinks (black) and incorrect crosslinks (grey) identified with an estimated CSM-FDR of 5%. **d** Overlap of correct (left) and incorrect crosslinks (right) for one technical repeat. Values for figures **a** and **c** are given in Supplementary Tables 2 and 3, and average values ± standard deviation are shown on the stacked bar plots (rounded to the closest whole number). All results files can be found in Supplementary Data 1. Error bars correspond to the standard deviation between technical replicates (*n* = 3).

The DSS-crosslinked peptide library was measured via LC-MS/MS and the resulting data were analysed with pLink, StavroX and Xi (Fig. 2). Figure 2a shows the number of crosslink spectrum matches (CSMs) identified by the different search algorithms that correspond to correct (black) and incorrect (grey) crosslinks. To assess the reproducibility of XL-MS workflows, values are presented as an average of three technical replicates, and the error bars represent the standard deviation. For all algorithms, the data were searched against the sequence of *S. pyogenes* Cas9 and 10 additional proteins (Supplementary Data 2) and the results were filtered to an estimated 5% FDR at the CSM level (referred to from here on as CSM-FDR).

pLink identifies the highest number of correct CSMs (678) and has a calculated FDR of 4.3%. StavroX and Xi identify a lower number of correct CSMs (410 and 512 respectively), with lower calculated FDR values of 2.4% and 2.5% respectively. The Venn diagrams display the overlap of scans from which CSMs are derived by the three algorithms, corresponding to correct (left) and incorrect (right) crosslinks. Out of 682 spectra that are matched to correct crosslinks by at least 1 search engine in the first technical repeat, 275 are matched by all 3, and almost all the spectra (639) are matched by pLink. Out of 45 scans that are matched with incorrect crosslinks by at least one algorithm, 27 are matched by pLink. One scan is matched to the same, incorrect crosslink by all three algorithms. Upon manual inspection of the spectrum, we found that the incorrect isotope peak is assigned to the mass of the precursor by the mass spectrometer.

The number of correct and incorrect unique crosslinks identified by the three algorithms at an estimated 5% CSM-FDR are shown in Fig. 2c. In the case of all algorithms, the calculated FDR of the unique crosslinks is higher than that of the CSMs. While the calculated FDR of the CSMs identified by pLink is 4.3%, this is propagated to 11.3% in the case of the unique crosslinks. This is due to CSM redundancy. For the correct crosslinks there is an average of 2.9 CSMs per crosslink. For the incorrect crosslinks, this ratio is 1.0. This effect is similar for StavroX and Xi that have CSM redundancies of 2.4 and 2.7, respectively. Out of 221 crosslinks that are identified by at least 1 search engine in the first technical repeat, 217 are identified by pLink, 179 are identified by Xi and 159 are identified by StavroX (Fig. 2d).

The number of CSMs and crosslinks identified when the results are filtered to an estimated CSM-FDR of 1% are shown in Supplementary Fig. 1, and values given in Supplementary Tables 4 and 5. Calculated FDR values of the unique crosslinks are 6.6%, 1.1% and 1.6% for pLink, StavroX and Xi, respectively, with pLink identifying 207 correct crosslinks, StavroX identifying 102 and Xi identifying 152.

### Effect of validation strategies on crosslink results

**Fig. 3:**
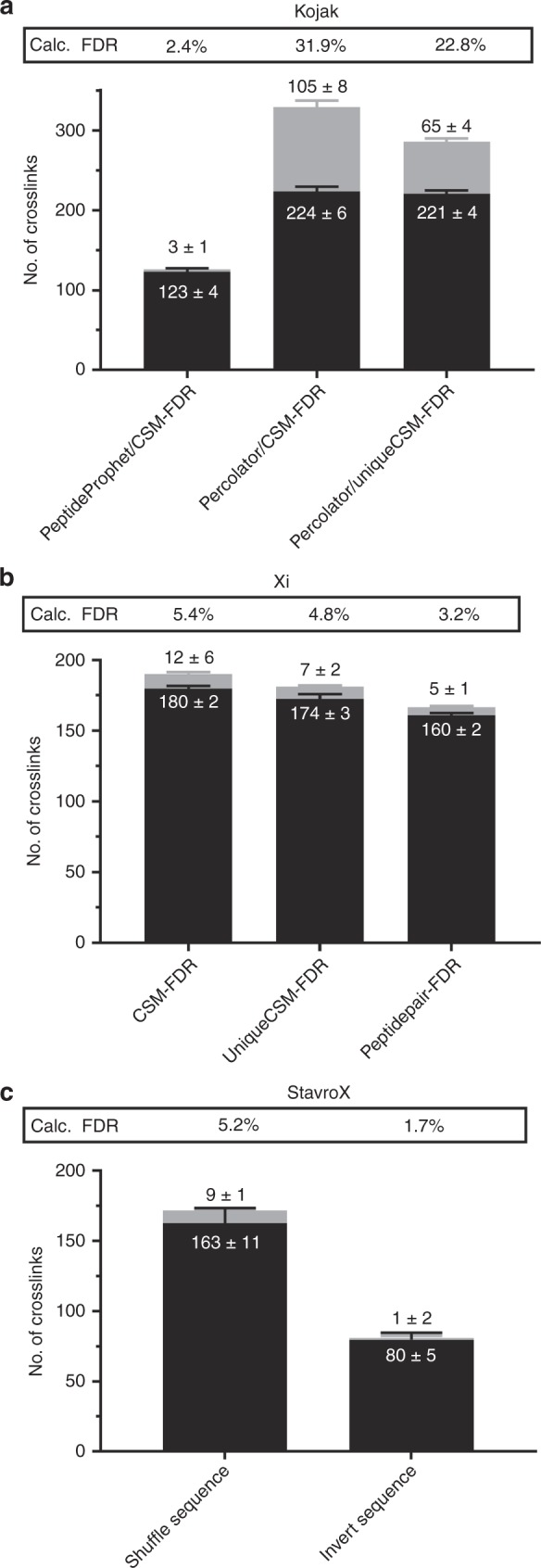
Effect of crosslink validation strategies on overall results. **a** Kojak results were validated by PeptideProphet or Percolator with which all CSMs were used, or only the top-scoring CSM for each species. **b** Xi results were validated at the CSM-FDR level, the unique CSM-FDR level or at the peptide pair-FDR level. **c** StavroX results were validated against a decoy database in which the sequences were shuffled with the protease sites remaining unchanged, or in which the sequences were inverted. All values are provided in Supplementary Table 6, and the average values ± standard deviation are shown on the stacked box plots. All results files can be found in Supplementary Data 1. Error bars correspond to the standard deviation between technical replicates (*n* = 3).

The effect of different validation methods on the sensitivity and accuracy of crosslink assignment is shown in Fig. 3. In all cases, the results were filtered to an estimated FDR of 5%, and the calculated FDR is reported in terms of unique crosslinks. We started by validating Kojak results with different tools, namely PeptideProphet^19^ and Percolator^18^ (Fig. 3a), since Kojak has no built-in validation method. We observed that PeptideProphet, which estimates FDR at the CSM level, is a very stringent validation method that has a calculated FDR of 2.4%, and that sensitivity is very low compared to other algorithms; only 123 correct crosslinks were identified. Percolator was used for validation by considering the CSM-FDR, or by considering only the highest-scoring CSM for each species (unique CSM-FDR). Both methods allow a much higher degree of sensitivity, but this comes with the price of much lower precision. When results are filtered at the CSM-FDR level, 224 correct crosslinks were identified, which is the highest of all identification approaches, but with a highly elevated calculated FDR of 32%. At the unique CSM-FDR, 221 correct crosslinks were identified with a slightly lower calculated FDR of 23%.

Fischer et al.^9^ report that FDRs can be estimated at different points during data analysis, and it is implemented into Xi that the FDR can be calculated on different levels. We compared FDR estimations calculated at the CSM-FDR level, the unique CSM-FDR level and the peptide pair-FDR level (Fig. 3b). We note a slight decrease in the number of crosslinks identified when the FDR calculation is performed later in the analysis (180, 174 and 160), along with a decrease in calculated FDR (5.4%, 4.8% and 3.2%) for the CSM level, unique CSM level and peptide pair level, respectively. The distribution of scores attributed to target and decoy sequences by Xi during FDR calculations at different levels can be found in Supplementary Fig. 2.

StavroX offers two possibilities for generating the decoy database: to shuffle sequences and keep protease sites or to invert the sequences. Crosslink validation with an inverted sequence is much more stringent than with the ‘shuffled sequence/ same protease sites’ option. While 163 crosslinks are found with the former (calculated FDR 5.2%), just 80 crosslinks are identified with the latter, with a calculated FDR of 1.7% which is much lower than the estimated CSM-FDR of 5%.

We were also interested to see how the various search algorithms perform when the data are searched against a larger database (Supplementary Fig. 3, Supplementary Table 7). For this we used a database containing the sequence of *S. pyogenes* Cas9 and 116 contaminant proteins (the crapome^27^). For pLink and the Kojak/Percolator combination, the larger database allowed a more accurate CSM-FDR estimation, along with a slight reduction in the number of correct crosslinks being identified. For pLink the calculated FDR is reduced from 11.3% to 6.1% and the number of correct crosslinks is reduced from 217 to 204, while for Kojak/Percolator the calculated FDR when estimated at the CSM level is reduced from 23% to 12% with a reduction in correct crosslinks from 221 to 202. Interestingly, searching against the larger database resulted in more crosslinks being identified with Xi (e.g. from 160 to 175 for the peptide pair level) along with an increase in calculated FDR (e.g. 3.2% to 6.7% for the peptide pair level). For the Kojak/PeptideProphet combination, the larger database increases the FDR (2.4–3.7%) while reducing the number of correctly identified crosslinks (123–121), both of which are undesirable effects in a crosslinking study.

### Comparison of scores attributed to CSMs

**Fig. 4:**
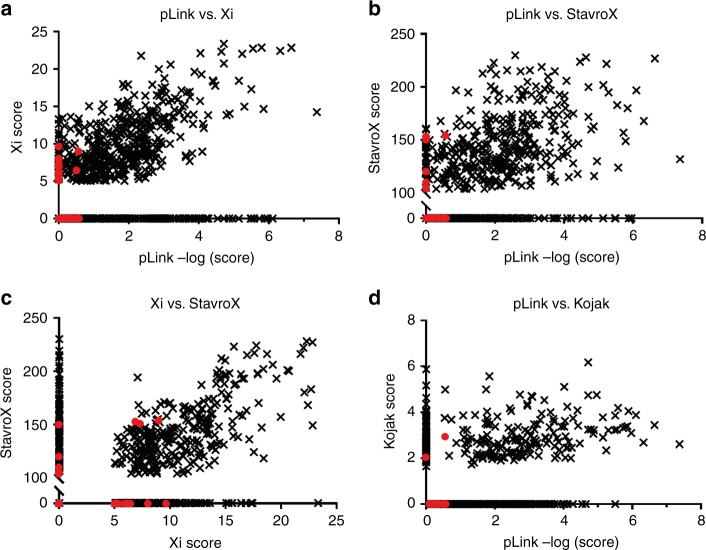
Comparison of scores assigned to each CSM by pLink, StavroX, Xi and Kojak. CSMs correlating to correct crosslinks are shown in black, and CSMs correlating to false positives are shown in red. **a** pLink vs. Xi, **b** pLink vs StavroX, **c** Xi vs StavroX, and **d** pLink vs Kojak.

Another possible reason for the discrepancy between algorithms is the scoring functions. To investigate this further, scores attributed to CSMs by the various algorithms are compared (Fig. 4). pLink scores given to each CSM are compared with those given by Xi (Fig. 4a), StavroX (Fig. 4b) and Kojak (Fig. 4d), and scores given by Xi are compared to those given by StavroX (Fig. 4c). CSMs correlating to correct crosslinks are shown in black, and CSMs correlating to false positives are shown in red. The scores attributed by pLink to false-positive identifications are at the lower end of the scoring scale, suggesting that the pLink scoring system is robust. It is therefore expected that a slightly more stringent scoring approach would remove the false positives with little effect on the true crosslinks. There is positive correlation between the scores of pLink and Xi (*R*^2^ = 0.41) and the scores of StavroX and Xi (*R*^2^ = 0.44), with lower correlation between pLink and StavroX (*R*^2^ = 0.15) and even lower between pLink and Kojak (*R*^2^ = 0.06). This demonstrates that all algorithms have very different approaches for scoring crosslink assignments.

### Score distributions for candidate and decoy CSMs

**Fig. 5:**
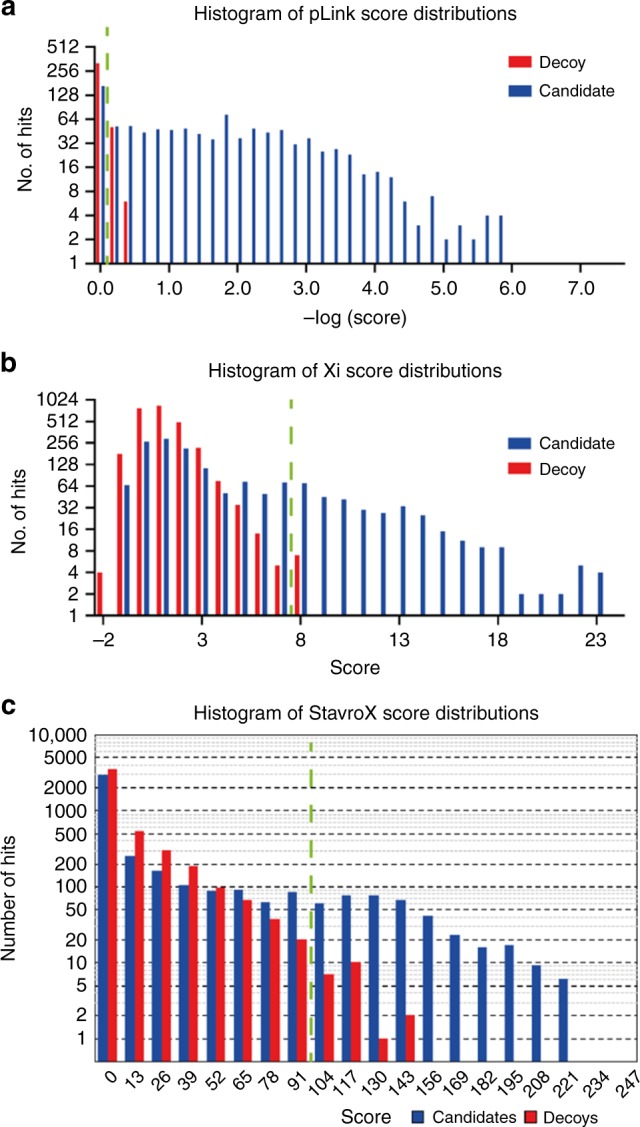
Score distributions for candidate and decoy crosslinks given by search algorithms. **a** pLink, **b** Xi and **c** StavroX. Here, the true-positive and false-positive crosslinks are grouped as candidates. Green dashed line refers to the score cut-off used to filter for an estimated 5% FDR.

The score distributions for the candidate and decoy peptides are shown for pLink (Fig. 5a), Xi (Fig. 5b) and StavroX (Fig. 5c). The distribution of the scores given to decoy crosslinks correlates well with the stringency and accuracy of the algorithms. pLink attributes very low scores to the decoy crosslinks, and the score cut-off for the candidate crosslinks is therefore very low. StavroX on the other hand attributes a range of scores to decoy peptides, and the cut-off value for candidate crosslinks is very high, providing a stringent validation. For Xi, the situation lies between the two extremes; the number of correct and incorrect crosslinks lies between the number found for pLink and StavroX.

### Number of MS2 spectra utilised by the search engines

**Fig. 6:**
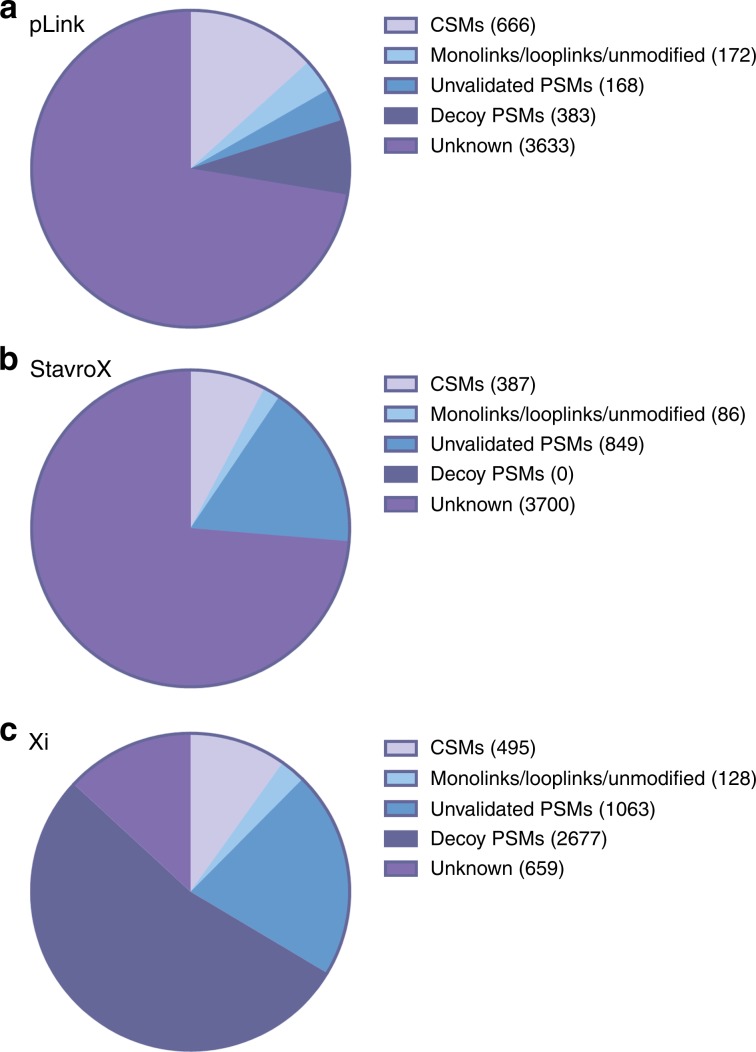
number of MS2 spectra that are attributed to specific features. **a** pLink, **b** StavroX and **c** Xi. Shown is the number of MS2 spectra annotated as CSMs, monolinks/looplinks/unmodified peptides, unvalidated PSMs with a score below the cut-off value for 5% FDR, decoy PSMs, and PSMs for which no information is known (unknown).

During measurement of the first technical replicate of the DSS-crosslinked peptides, 5022 MS2 spectra were triggered during the MS acquisition. In the case of pLink, 666 of these were assigned to crosslinks, 172 were assigned to monolinks, looplinks and unmodified peptides, 168 were assigned to candidate crosslinks that had a score below the cut-off for the estimated 5% CSM-FDR, 383 were assigned to decoy PSMs (corresponding to all species) and 3633 triggered spectra were not utilised in the search (Fig. 6). From the 168 spectra that had a score below the cut-off value for 5% CSM-FDR, just 4 additional correct crosslinks were identified that failed to pass the validation. An additional 105 incorrect crosslinks were found in this ‘unvalidated’ portion of the CSMs, demonstrating efficient separation of crosslinks that are correctly and incorrectly assigned.

StavroX does not provide information on unmodified peptides or the spectra that were assigned to decoy crosslinks. Nevertheless, a much higher number of spectra were assigned to unvalidated species that had a score below the cut-off for an estimated 5% CSM-FDR than pLink. From these, an additional 58 correct crosslinks were identified, and 389 incorrect. This suggests that StavroX does not have a problem with identifying crosslinks, but rather has difficulty distinguishing those that are correct from those that are incorrect.

Xi ascribes 2677 spectra to decoy species, much higher than pLink (383), which is a potential reason for it being a more stringent search algorithm. Eleven additional correct crosslinks can be identified in the ‘non-validated’ CSMs, which is a much lower number than for StavroX, while 757 additional incorrect crosslinks are identified, which is more than both StavroX and pLink. Xi therefore appears to have a robust method of separating correct crosslinks from incorrect.

### Assessment of workflows developed for cleavable crosslinkers

**Fig. 7:**
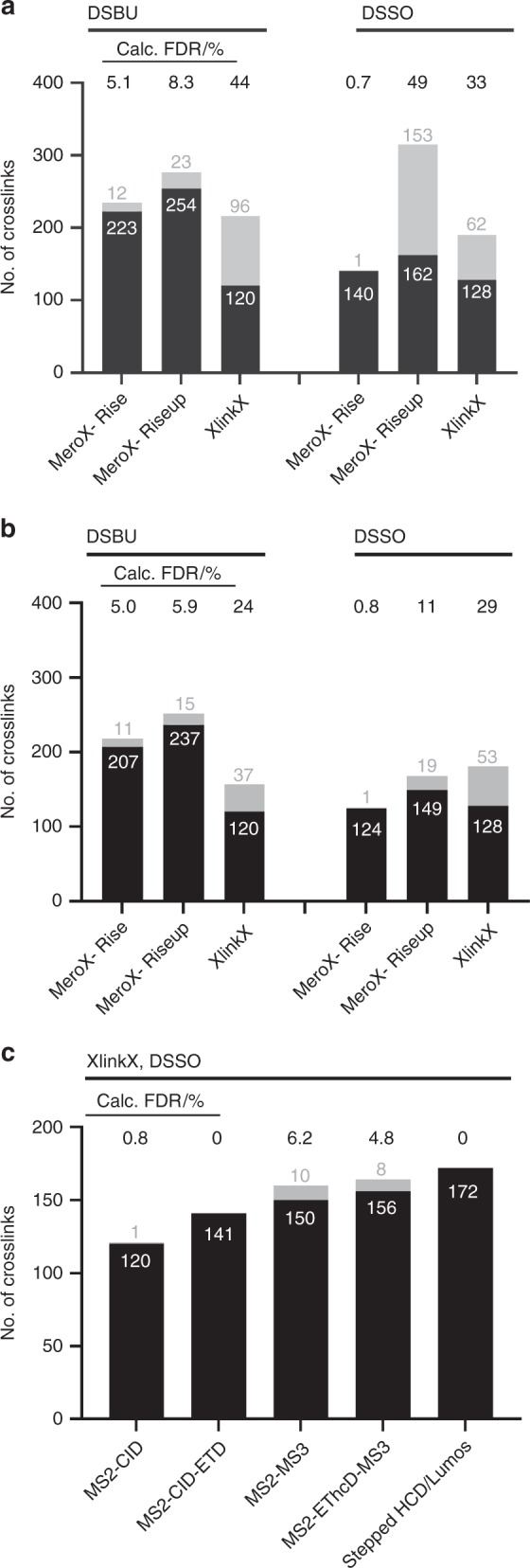
Assessment of algorithms for the identification of peptides crosslinked with cleavable reagents DSBU and DSSO. **a**, **b** Data were collected using MS2 method with stepped collision energy, analysed using MeroX (in Rise and RiseUP mode) and XlinkX, with data filtered to an estimated 5% FDR (**a**) and 1% FDR (**b**), with no additional score cut-off values employed. **c** Data for DSSO-crosslinked peptides were collected using HCD-, MS3- and ETD-based methods, and analysed using XlinkX with the recommended score cut-off values of 45 and 4 for crosslink score and Δcrosslink score, respectively. Values are given in Supplementary Tables 8–10.

Recently, MS-cleavable crosslinkers have been developed to increase confidence of crosslink assignments and to allow identification of crosslinks in more complex samples. Here, the peptides were crosslinked as before with DSBU or DSSO and measured using stepped HCD on an Orbitrap Q-Exactive HF-X (Fig. 7). Data are searched against a database containing the sequence of *S. pyogenes* Cas9 and the crapome^27^ with MeroX 2.0 (in Rise and RiseUP mode)^28^ and XlinkX in Proteome Discoverer 2.3 (ref. ^29^). This larger database was used for analysis of peptides crosslinked with the cleavable reagents compared to DSS, because a key benefit of such crosslinkers is the ability to search data against larger databases, up to the scale of the human proteome. Also, it has been recommended by the developers to use a database with at least 100 other protein sequences that are used as support for FDR control^29^.

We first analysed data recorded on a Q-Exactive HFX with a stepped HCD fragmentation method as recommended by Iacobucci et al.^28^. Data were filtered to an estimated CSM-FDR of 5% with no additional score cut-off values. When data from the measurement of DSBU peptides are analysed by MeroX in Rise mode (Fig. 7a), in which three out of four reporter peaks must be present in the MS2 spectrum, 223 correct crosslinks and 12 incorrect crosslinks are identified, giving rise to a calculated FDR of 4.9%. When used in RiseUP mode, which appears to be a less stringent analysis method, the number of correct crosslinks increases to 254 but the number of false identifications also rises to 23 resulting in an increased calculated FDR of 8.3%. XlinkX finds 120 correct crosslinks and 96 false positives, resulting in a calculated FDR of 44%. One reason that fewer crosslinks are detected with XlinkX is that, unlike MeroX, it does not report crosslinks between two peptides of the same sequence. When such crosslinks are removed from the MeroX search results, the number of correctly identified crosslinks are 169 and 199 for Rise and RiseUP mode, respectively. MeroX identifies a lower number of DSSO-crosslinked peptides than DSBU, likely because MeroX was developed for the analysis of DSBU-crosslinked samples. In Rise mode 140 true crosslinks are identified (with 1 false positive, calculated FDR 0.7%), and in RiseUP mode 162 true crosslinks are identified (153 false positives, calculated FDR 49%). XlinkX identifies 128 true DSSO crosslinks (with 62 false positives, calculated FDR 33%), which is 8 more than were identified with DSBU. When the estimated CSM-FDR cut-off was reduced to 1%, the calculated FDR for the DSBU/Rise combination actually increased to 5%, the DSBU/Riseup combination reduced to 5.9% and the DSBU/XlinkX combination reduced to 24%. For the DSSO-crosslinked peptides the calculated FDR is reduced to 0.8% for Rise, 11% for Riseup and 29% for XlinkX. We were surprised to note from this study that the estimated FDR is often less reliable for cleavable crosslinkers than can be achieved with DSS (Fig. 2).

Methods involving MS3 and ETD that were developed for the measurement of DSSO-crosslinked peptides on an Orbitrap Fusion Lumos were also investigated, and the data were analysed using XlinkX (Fig. 7c). Here, we also employed the recommended score cut-off of 45 and minimum score difference of 4 (ref. ^29^). Upon measurement with CID-ETD, 141 crosslinks were identified, all of which were correct. MS2–MS3 measurements allowed identification of 150 correct and 10 incorrect crosslinks, resulting in a calculated FDR of 6.2%. The hybrid method MS2-EThcD-MS3 gives rise to 156 correct and 8 incorrect crosslinks (calculated FDR 4.9%). Measurement with stepped HCD gives rise to the highest number of true crosslinks (172) with just one false positive (0.5% calculated FDR).

## Discussion

This unique synthetic crosslinked peptide library is an excellent resource for the crosslinking community. The data arising from analysis of the crosslinked peptides can be used for in-depth assessment of the many algorithms available for XL identification. This provides valuable information for users of XL-MS regarding the performance of crosslink search algorithms, allowing them to select data analysis strategies that meet their needs in terms of confidence and sensitivity of crosslink assignments. The data will also be invaluable in the development of crosslink search engines, since it is known which crosslinks can be true, and which are falsely identified. This allows better assessment of different FDR calculations that are currently available.

This study also allows assertions to be made about the analysis of crosslinking data. Eminently, when using pLink, or the Kojak/Percolator combination, a large database is required for better FDR estimation. A reason for better performance with the larger database could be the higher number of CSMs in the resulting set that increase the training size for the algorithm, or it could be that false CSMs are less likely to be randomly assigned to Cas9, thereby producing a more defined decoy population exploited by the algorithms during the training regimen. This is particularly likely to be true for Percolator that performs best when trained on 100,000 or more spectra^18^, which is far more than the 5022 contained in this dataset. Alternatively, in our study, Xi and Kojak/PeptideProphet perform better with a smaller database, for example with 10 proteins. The data included in this publication can be utilised to further optimise such parameters.

Additionally, when using XlinkX for the analysis of data in which cleavable crosslinkers were used, the score cut-offs are important in filtering for high-quality data that more likely allow correct crosslink identification. One should be aware that while such cut-offs work well in this study and others^30,31^, they give no indication of the confidence in crosslink assignment. Judging by the data, different cut-off values could be optimised for different fragmentation techniques. For example, a lower value of 20 could be used for the stHCD data that gives rise to 183 correct crosslinks (11 more than with a score cut-off of 40) and 8 incorrect, resulting in a calculated FDR of 4.1%. A similar approach was taken by Ser et al.^5^ by spiking crosslinked BSA peptides into non-crosslinked proteome background peptides to calculated score filters that removed 99% and 90% of non-BSA crosslinks to determine FDR cut-offs for 1% and 10% FDR, respectively. The data included in the present publication could be even more useful for a systematic optimisation of search and validation strategies.

Results from XL-MS studies have far-reaching implications, often leading to new avenues of research regarding novel protein–protein interactions. It is therefore of paramount importance that the confidence of identified crosslinks is correctly estimated. As well as providing a useful bioinformatics resource, the physical library can be used to optimise many stages of the crosslinking workflow including crosslink enrichment strategies, chromatography methods and MS data acquisition parameters.

## Methods

### Peptide design and synthesis

Peptides were synthesised on a SYRO with Tip Synthesis Module (MultiSynTech GmbH) using standard Fmoc chemistry. For each amino acid cycle, double coupling with DIC/K-Oxyma and HATU/DIEA was performed. C-terminal lysine residues were initially incorporated with an azide group. Peptides were purified on a C18 kinetex column (2.6 µm) using a 30 min gradient, and the identity of the peptides was confirmed using MALDI-MS (4800 MALDI TOF/TOF, Applied Biosystems). Peptide concentration was measured using a nanodrop, the solution was evaporated to almost dryness, and the peptides were resuspended at a concentration of 5 mM in HEPES buffer (100 mM pH 8) and the appropriate peptides were combined into groups (Supplementary Table 1).

### Crosslinking

Crosslinker was dissolved at 20 mM in DMSO, and 0.5 µl were added to 5 µl to each peptide group 5× over the course of 2.5 h. One microliter of each crosslinked peptide group was added to 9 µl ammonium bicarbonate (ABC, 100 mM) for overnight digestion with 10 ng trypsin at 37 °C and subsequent reduction with tris(2-carboxyethyl)phosphine for 30 min (final concentration 50 mM). All 12 groups were combined into one sample which was stored at −80 °C in 5 µl aliquots for future use.

### Biotin removal

After digestion, the biotin-linker region was removed with streptavidin beads. Twenty-five microliters of slurry was washed three times with 25 µl ABC buffer. In all, 5 µl peptides + 20 µl ABC buffer was loaded onto the beads and incubated for 30 min. The eluate was removed and the beads were washed with 25 µl 0.1% FA and subsequently 25 µl 30% MeOH. The eluates were combined and reduced to 5 µl in a centrifugal concentrator to remove the MeOH. The sample was then diluted 5× in 0.1% TFA and 1 µl of the final solution was injected.

### Reversed-phase high-performance liquid chromatography

Crosslinked peptides were separated using a Dionex UltiMate 3000 high-performance liquid chromatography (HPLC) RSLCnano System prior to MS analysis. The HPLC was interfaced with the mass spectrometer via a Nanospray Flex ion source. For sample concentrating, washing and desalting, the peptides were trapped on an Acclaim PepMap C-18 precolumn (0.3 × 5 mm, Thermo Fisher Scientific) using a flow rate of 25 μL/min and 100% buffer A (99.9% H_2_O, 0.1% TFA). The separation was performed on an Acclaim PepMap C-18 column (50 cm × 75 μm, 2 μm particles, 100 Å pore size, Thermo Fisher Scientific) applying a flow rate of 230 nL/min. For separation, a solvent gradient ranging from 2% to 40% buffer B (80% ACN, 19.92% H_2_O, 0.08% TFA) was applied. The gradient length was 2 h for the analysis of the combined groups.

### Mass spectrometry

MS settings were used according to previously published protocols. DSSO- and DSBU-crosslinked peptides were measured on both an Orbitrap Q-Exactive HF-X instrument and an Orbitrap Fusion Lumos mass spectrometer. DSS samples were measured on an Orbitrap Q-Exactive HF-X instrument with the settings recommended by Chen and Rappsilber^16^ (MS1 Orbitrap resolution 120K, MS1 *m*/*z* scan range 400–1600, MS1 maximum injection time 20 ms, MS1 AGC target 2e5, dynamic exclusion 30 s with 10ppm mass window, minimum precursor intensity 5e4, charge state exclusion <3+ or >8+, isolation window 2*m*/*z*, normalised collision energy 28, TopN *N* = 10 starting with the most intense MS1 signal, MS2 resolution 35 K, MS2 maximum injection time 60 ms, MS2 AGC target 1e5) DSBU measurements on the HFX were measured with the settings recommended by Iacobucci et al.^28^. (MS1 orbitrap resolution 140K, MS1 *m*/*z* scan range 300–1700, MS1 maximum injection time 100 ms, MS1 AGC target 3e6, dynamic exclusion 60 s with 2 ppm mass window, charge state exclusion <3+ or >8+, isolation window 2*m*/*z*, stepped HCD with normalised collision energies 27%, 30% and 33%, TopN *N* = 10 starting with the most intense MS1 signal, MS2 resolution 17.5K, MS2 maximum injection time 250 ms, MS2 AGC target 2e5). DSSO samples were measured on an Orbitrap Fusion Lumos mass spectrometer with the settings recommended by Klykov et al.^29^ for MS3 and ETD-based acquisitions (MS1 orbitrap resolution 60K, MS1 *m*/*z* scan range 375–1500, MS1 AGC target 4e5, MS1 maximum injection time 50 ms, charge state exclusion <3+ or >8+, dynamic exclusion 30 s with 10 ppm mass window. MS2-CID: CID collision energy 30%, MS2 resolution 30K, AGC target 5e4, maximum ion injection time 100 ms. MS2-ETD: MS2 resolution 30K, AGC target 1e5, maximum ion injection time 120 ms. MS3: MS2 isolation window 2*m*/*z*, collision energy 35%, AGC target 3e4, maximum ion injection time 90 ms). Settings recommended by Stieger et al.^32^ were used for stepped HCD methods (MS1 orbitrap resolution 60K, MS1 *m*/*z* scan range 375–1500, MS1 maximum injection time 50 ms, MS1 AGC target 5e5, dynamic exclusion 30 s with 10 ppm mass window, charge state exclusion <3+ or >8+, isolation window 1.6*m*/*z*, stepped HCD with normalised collision energies 21%, 27% and 33%, TopN *N* = 10 starting with the most intense MS1 signal, MS2 resolution 30K, MS2 maximum injection time 100 ms, MS2 AGC target 5e4).

### Data analysis

Raw data collected from the measurement of DSS-crosslinked peptides were converted to.mgf format using MSconvert with the Peak Picking function and searched with the relevant programmes (pLink 2.3.5, StavroX 3.6.0, Xi 1.6.751, kojak 1.6.1) against a database containing *S. pyogenes* Cas9 and 10 additional proteins (Supplementary Data 2). Settings for each programme are given in Supplementary Table 11. Data collected from the measurement of cleavable crosslinkers were searched with MeroX 2.0 beta 5 and XlinkX in proteome discoverer 2.3 against a database containing *S. pyogenes* Cas9 and the Crapome^27^. Settings are given in Supplementary Information Table 12, and all search outputs are provided in Supplementary Data 1.

### Reporting summary

Further information on research design is available in the Nature Research Reporting Summary linked to this article.

## Supplementary information

Supplementary Information

Description of Additional Supplementary Files

Supplementary Data 1

Supplementary Data 2

Reporting Summary

## Data Availability

The mass spectrometry proteomics data have been deposited to the ProteomeXchange Consortium via the PRIDE^33^ partner repository with the dataset identifier PXD014337. All other data are available from the corresponding authors upon reasonable request.
