# Supplementary material for: A synthetic peptide library for benchmarking crosslinking-mass spectrometry search engines for proteins and protein complexes
Source: Nat Commun. 2020 Feb 6;11:742. doi: 10.1038/s41467-020-14608-2 (PMC7005041; doi:10.1038/s41467-020-14608-2)
Supplement: Supplementary file 1 — Supplementary Information [file 41467_2020_14608_MOESM1_ESM.pdf]

Supplementary Information:

Supplementary Table 1 - peptide sequences in each group.

Supplementary Table 2 - Number of CSMs attributed to DSS crosslinks by pLink, StavroX and Xi. Results were filtered to an estimated 5% FDR.

Supplementary Table 3 - Number of DSS crosslinks identified with pLink, StavroX and Xi. Results were filtered to an estimated 5% FDR.

Supplementary Figure 1 - Number of CSMs/ crosslinks identified by pLink, StavroX and Xi. Results were filtered to an estimated 1% FDR

Supplementary Table 4 - Number of CSMs attributed to DSS crosslinks by pLink, StavroX and Xi. Results were filtered to an estimated 1% FDR.

Supplementary Table 5 - Number of DSS crosslinks identified with pLink, StavroX and Xi. Results were filtered to an estimated 1% FDR.

Supplementary Table 6 - Number of DSS crosslinks identified with Kojak, Xi and StavroX employing different validation strategies, as shown in Figure 3.

Supplementary Figure 2 – The distribution of scores attributed to target and decoy sequences by Xi in FDR estimations at different levels

Supplementary Figure 3 - Number of DSS crosslinks identified when the data were searched against the Cas9 sequence and the CrapDB

Supplementary Table 7 - Number of DSS crosslinks identified when the data were searched against the Cas9 sequence and the CrapDB

Supplementary Table 8 - Number of DSBU or DSSO crosslinks identified with MeroX or XlinkX upon analysis of data generated with stepped HCD on a Q-exactive HFX instrument. Results were filtered to an estimated 5% FDR

Supplementary Table 9 - Number of DSBU or DSSO crosslinks identified with MeroX or XlinkX upon analysis of data generated with stepped HCD on a Q-exactive HFX instrument. Results were filtered to an estimated 1% FDR

Supplementary Table 10; Number of crosslinks identified with XlinkX upon analysis of the data generated with different fragmentation strategies.

Supplementary Table 11 - Search settings used for the identification of DSS- crosslinked peptides.

Supplementary Table 12; Search settings used for the identification of DSBU- and DSSO-crosslinked peptides.

Group	Peptide sequences
**Group 1**	SDKNR<br>KLINGIR<br>KFDNLTK<br>FIKPILEK<br>APLSASMIKR<br>NPIDFLEAKGYK<br>LPKYSLFELENGR<br>TEVQTGGFSKESILPK
**Group 2**	VKYVTEGMR<br>FDNLTKAER<br>DFQFYKVR<br>YDENDKLIR<br>MIAKSEQEIGK<br>HKPENIVIEMAR<br>TILDFLKSDGFANR<br>KIECFDSVEISGVEDR<br>YVNFLYLASHYEKLK
**Group 3**	LSKSR<br>DKPIR<br>KDLIIK<br>MKNYWR<br>KGILQTVK<br>NSDKLIAR<br>DDSIDNKVLTR
**Group 4**	KLVDSTDK<br>IEKILTFR<br>KAIVDLLFK<br>VLSAYNKHR<br>IEEGIKELGSQILK<br>SSFEKNPIDFLEAK<br>SNFDLAEDAKLQLSK<br>HSLLYEYFTVYNELTKVK
**Group 5**	KVTVK<br>EKIEK<br>VITLKSK<br>QLKEDYFK<br>QLLNAKLITQR<br>GGLSELDKAGFIK<br>MDGTEELLVKLNR
**Group 6**	EVKVITLK<br>KPAFLSGEQK<br>ENQTTQKGQK<br>KTEVQTGGFSK<br>VVDELVKVMGR<br>LESEFVYGDYKVYDVR<br>MLASAGELQKGNELALPSK<br>NFMQLIHDDSLTFKEDIQK<br>VLPKHSLLYEYFTVYNELTK

**Group 7**	KMIAK<br>ESILPKR<br>DLIIKLPK<br>FKVLGNTDR<br>SEQEIGKATAK<br>AIVDLLFKTNR<br>LKTYAHLFDDK<br>VNTEITKAPLSASMIK<br>YDEHHQDLTLLKALVR
**Group 8**	KDWDPK<br>QQLPEKYK<br>KVLSMPQVNIVK<br>MTNFDKNLPNEK<br>QITKHVAQILDSR<br>KSEETITPWNFEEVVDK<br>KNGLFGNLIALSLGLTPNFK<br>SKLVSDFR
**Group 9**	LKSVK<br>IIKDK<br>DWDPKK<br>LKGSPEDNEQK<br>VLSMPQVNIVKK<br>LENLIAQLPGEKK<br>LIYLALAHMIKFR<br>YPKLESEFVYGDYK
**Group 10**	VPSKK<br>VTVKQLK<br>EDYFKK<br>VKYVTEGMR<br>GKSDNVPSEEVVK<br>LEESFLVEEDKK<br>QEDFYPFLKDNR
**Group 11**	GQKNSR<br>AGFIKR<br>GYKEVK<br>VMKQLK<br>KDFQFYK<br>LVDSTDKADLR<br>SDNVPSEEVVKK<br>KNLIGALLFDSGETAEATR
**Group 12**	HSIKK<br>DKQSGK<br>NLPNEKVLPK<br>QSGKTILDFLK<br>MNTKYDENDK<br>SVKELLGITIMER<br>TYAHLFDDKVMK<br>FNASLGTYHDLLKIIK

Supplementary Table 1; peptide sequences in each group.

Search engine	Number of crosslink- spectrum matches								
	Correct			Incorrect			Calculated FDR (%)		
	R1	R2	R3	R1	R2	R3	R1	R2	R3
**pLink**	639	712	683	27	27	39	4.1	3.7	5.4
**StavroX**	378	434	419	9	12	10	2.3	2.7	2.3
**Xi**	491	498	547	20	13	10	3.9	2.6	1.8

Supplementary Table 2; Number of CSMs attributed to DSS crosslinks by pLink, StavroX and Xi. Results were filtered to an estimated 5% FDR. Measurements were performed in technical triplicate (R1, R2, R3).

Search engine	Number of crosslinks								
	Correct			Incorrect			Calculated FDR (%)		
	R1	R2	R3	R1	R2	R3	R1	R2	R3
pLink	217	230	203	26	24	33	10.7	9.4	14.0
StavroX	159	175	154	8	10	9	4.8	5.4	5.5
Xi	179	183	179	18	11	7	9.1	5.7	3.8

Supplementary Table 3; Number of DSS crosslinks identified with pLink, StavroX and Xi. Results were filtered to an estimated 5% FDR. Measurements were performed in technical triplicate (R1, R2, R3).

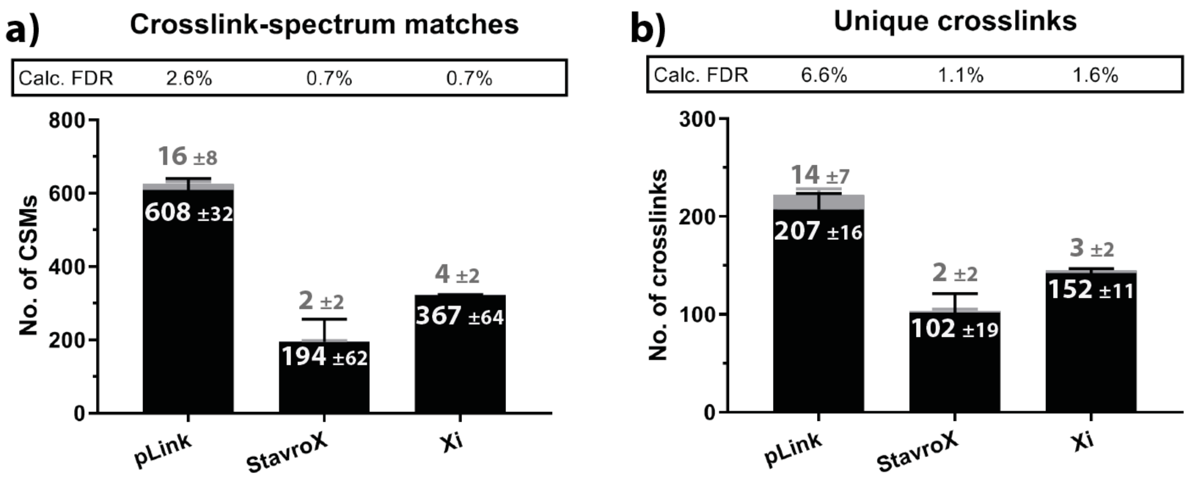

Supplementary Figure 1. (a) number of CSMs that correspond to correct (black) and incorrect (grey) crosslinks identified by pLink, StavroX and Xi. Results were filtered to an estimated 1% FDR, and the calculated FDR is given for each algorithm. Error bars correspond to the standard deviation between three technical replicates. (b) number of correct unique crosslinks (black) and incorrect crosslinks (grey). Values are given in Supplementary tables 4 and 5.

Search engine	Number of crosslink- spectrum matches								
	Correct			Incorrect			Calculated FDR (%)		
	R1	R2	R3	R1	R2	R3	R1	R2	R3
**pLink**	594	644	585	10	13	25	1.7	2.0	4.1
**StavroX**	265	157	160	4	0	1	1.5	0	0.6
**Xi**	312	352	438	2	4	5	0.6	1.1	1.1

Supplementary Table 4; Number of CSMs attributed to DSS crosslinks by pLink, StavroX and Xi. Results were filtered to an estimated 1% FDR. Measurements were performed in technical triplicate (R1, R2, R3).

Search engine	Number of crosslinks								
	Correct			Incorrect			Calculated FDR (%)		
	R1	R2	R3	R1	R2	R3	R1	R2	R3
pLink	215	218	189	9	12	22	4.0	5.2	11.6
StavroX	124	91	90	4	0	1	3.1	0	1.1
Xi	141	152	163	2	3	5	1.4	1.9	3.0

Supplementary Table 5; Number of DSS crosslinks identified with pLink, StavroX and Xi. Results were filtered to an estimated 1% FDR. Measurements were performed in technical triplicate (R1, R2, R3).

Search engine	Number of crosslinks								
	Correct			Incorrect			Calculated FDR (%)		
	R1	R2	R3	R1	R2	R3	R1	R2	R3
Kjk, PepProphet	128	121	120	2	3	4	1.5	2.4	3.2
Kjk, Perc, all	222	230	219	96	108	112	30.1	32.0	33.8
Kjk, Perc, unique	220	225	217	68	60	67	23.6	21.0	23.6
Xi, all CSMs	179	183	179	18	11	7	9.1	5.7	3.8
Xi, unique CSMs	176	175	170	6	10	6	3.3	5.4	3.4
Xi, peptide pair	162	161	158	6	4	5	3.6	2.4	3.1
StavroX, Shuffle	159	175	154	8	10	9	4.8	5.4	5.5
StavroX, invert	74	81	84	3	0	1	3.9	0	1.2

Supplementary Table 6; Number of DSS crosslinks identified with Kojak, Xi and StavroX employing different validation strategies, as shown in Figure 3. Results are filtered to an estimated 5% FDR.

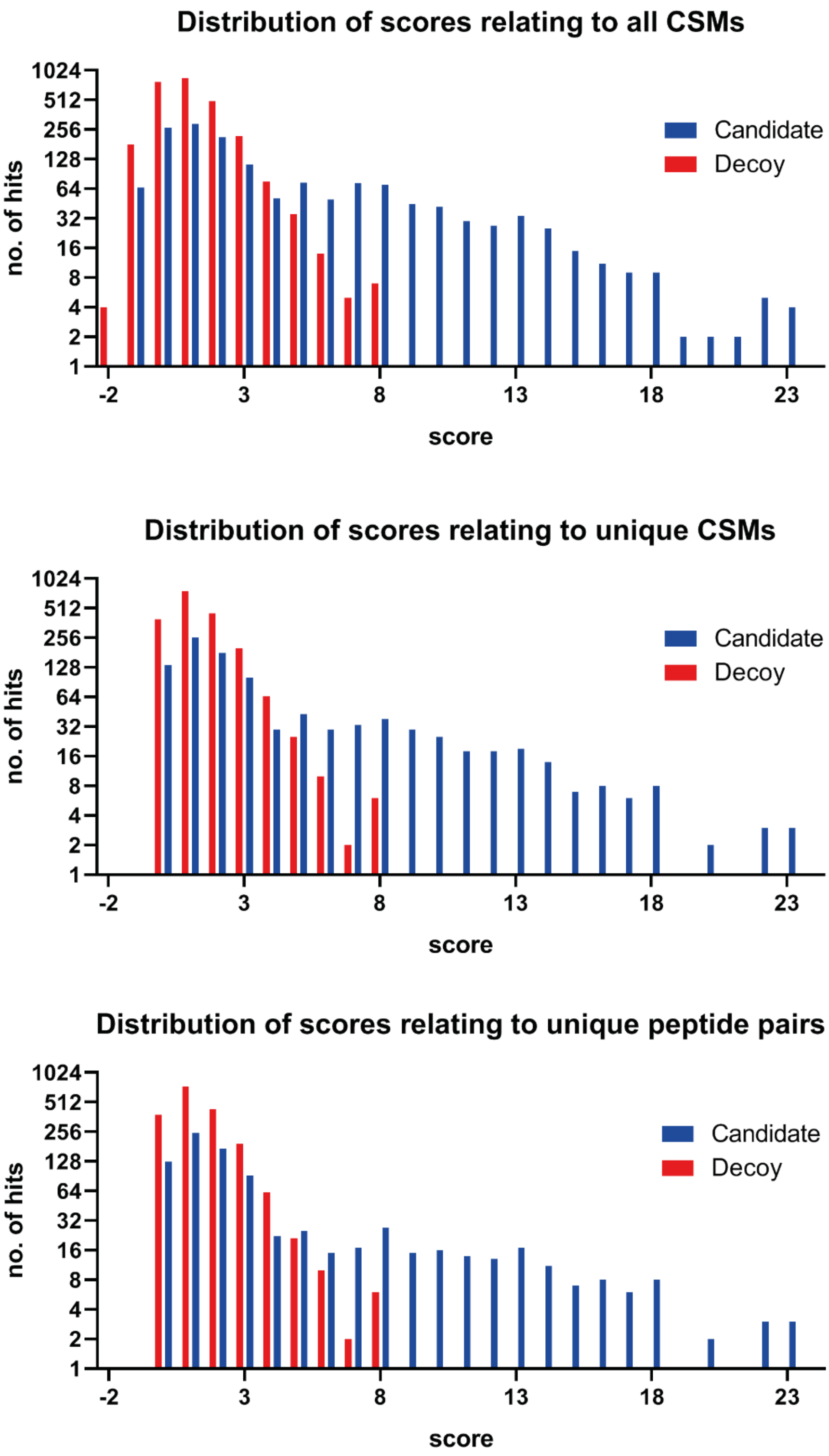

Supplementary Figure 2 The distribution of scores attributed to target and decoy sequences by Xi in FDR estimations at different levels. Score cut-off values for an estimated 5% FDR are 5.02, 5.6 and 6.4 at the CSM-FDR, the uniqueCSM-FDR and the peptidepair-FDR, respectively. The number of decoy crosslinks assigned above these thresholds are 28, 14 and 8.

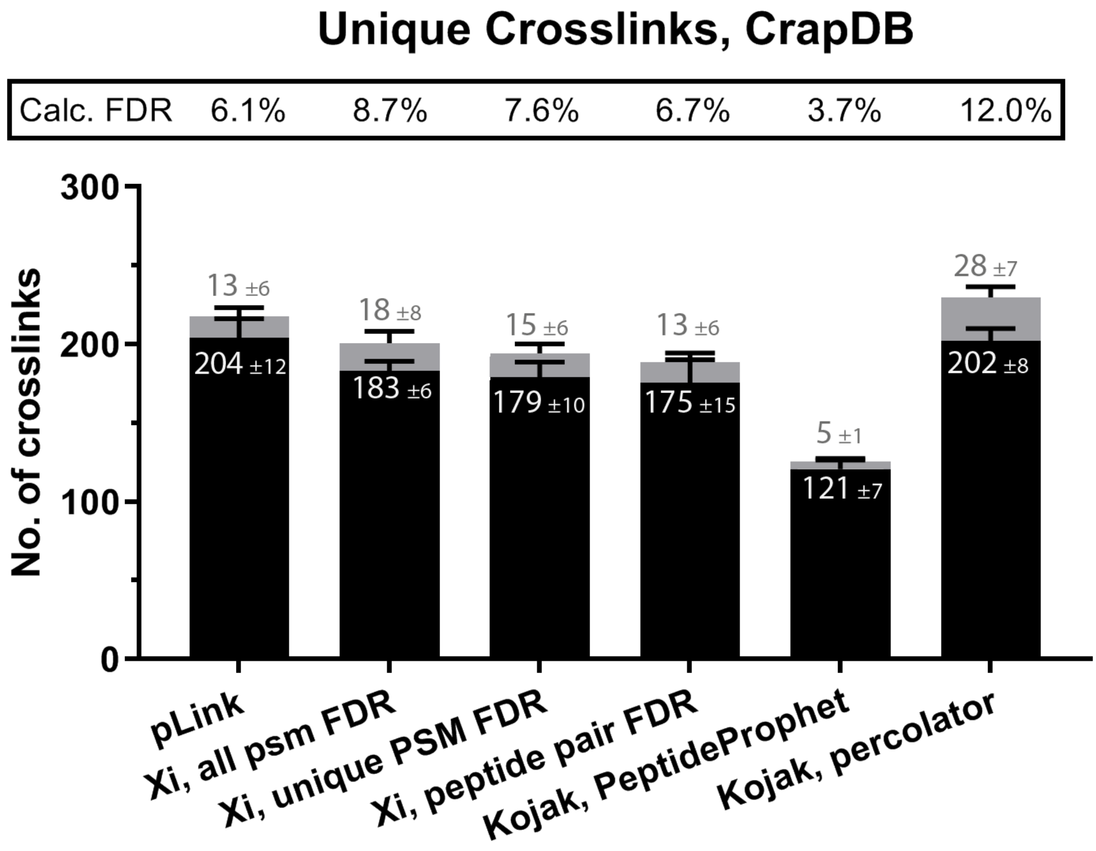

Supplementary Figure 3 Number of correct (black) and incorrect (grey) unique crosslinks identified when the data were searched against the Cas9 sequence and the CrapDB, which contains 116 proteins.

Search engine	Number of crosslinks								
	Correct			Incorrect			Calculated FDR (%)		
	R1	R2	R3	R1	R2	R3	R1	R2	R3
**pLink**	215	206	191	18	7	15	7.7	3.3	7.3
**Xi, all CSM**	181	190	178	22	22	9	10.8	10.4	4.8
**Xi, unique CSM**	180	188	169	19	18	8	9.5	8.7	4.5
**Xi, peptide pair**	179	188	159	17	16	6	8.7	7.8	3.6
**Kojak, PepProph**	126	113	123	4	4	6	3.1	3.4	4.7
**Kojak, Perc (unique)**	198	211	197	20	31	32	9.1	12.8	14.0

Supplementary Table 7; number of DSS crosslinks identified with pLink, Xi and Kojak when data were searched against the CrapDB, containing 116 proteins.

MS method	Number of crosslinks		
	Correct	Incorrect	Calculated FDR (%)
**DSBU, MeroX, Rise**	223	12	4.9
**DSBU, MeroX, Riseup**	254	23	8.3
**DSBU, XlinkX**	120	96	44
**DSSO, MeroX, Rise**	140	1	0.7
**DSSO, MeroX, Riseup**	162	153	49
**DSSO, XlinkX**	128	62	33

Supplementary Table 8; Number of DSBU or DSSO crosslinks identified with MeroX or XlinkX upon analysis of data generated with stepped HCD on a Q-exactive HFX instrument. Results were filtered to an estimated **5% FDR** with no extra score cut-offs implemented.

MS method	Number of crosslinks		
	Correct	Incorrect	Calculated FDR (%)
**DSBU, MeroX, Rise**	207	11	5.0
**DSBU, MeroX, Riseup**	237	15	5.9
**DSBU, XlinkX**	120	37	24
**DSSO, MeroX, Rise**	124	1	0.8
**DSSO, MeroX, Riseup**	149	19	11
**DSSO, XlinkX**	128	53	29

Supplementary Table 9; Number of DSBU or DSSO crosslinks identified with MeroX or XlinkX upon analysis of data generated with stepped HCD on a Q-exactive HFX instrument. Results were filtered to an estimated **1% FDR** with no extra score cut-offs implemented.

MS method	Number of crosslinks		
	Correct	Incorrect	Calculated FDR (%)
**MS2-CID**	120	1	0.08
**MS2-CID-ETD**	141	0	0
**MS2-MS3**	150	10	6.2
**MS2-EThcD-MS3**	156	8	4.8
**Stepped HCD, Lumos**	172	0	0

Supplementary Table 10; Number of crosslinks identified with XlinkX upon analysis of the data generated with different fragmentation strategies. Score cut-off values were implemented of 45 and 4 for crosslink score and Δcrosslink score, respectively.

	pLink (2.3.5)	StavroX (3.6.0)	Xi (1.6.751)	Kojak (1.6.1)
**Crosslink mass/ Da**	138.068	138.068	138.068	138.068
**Monolink mass/ Da**	156.079	156.079	156.079	156.079
**Crosslinker reactivity**	K-K	K-K	K-K	K-K
**Fixed modification**	Carbamido-methyl [C]	Carbamido-methyl [C]	Carbamido-methyl [C]	Carbamido-methyl [C]
**Variable modification**	Oxidation [M]	Oxidation [M]	Oxidation [M]	Oxidation [M]
**Enzyme**	Trypsin	Trypsin	Trypsin	Trypsin
**Max. missed cleavages**	3	R:3 K:3	3	3
**Min peptide mass**	500	500	-	500
**Max peptide mass**	6000	6000	-	6000
**Min peptide length**	5	5	5	-
**Max peptide length**	60	-	-	-
**MS1 tolerance (ppm)**	5	5	5	5
**MS2 tolerance (ppm)**	20	20	20	Bin size 0.03 Thomson
**FDR calculation**	inbuilt	inbuilt	Xi FDR (1.1.27)	Percolator (3.02)
**FDR level**	PSM	PSM	PSM	PSM

Supplementary Table 11; Search settings used for the identification of DSS- crosslinked peptides.

	XlinkX in proteome discoverer 2.3	MeroX 2.0 beta 5
**DSBU Crosslink mass/ Da**	196.085	196.085
**Bu-fragment/ Da**	-	85.053
**BuUr-fragment/ Da**	-	111.032
**DSSO crosslink mass/ Da**	158.004	158.004
**Alkene/ Da**	-	54.011 (essential)
**Thiol/ Da**	-	85.983 (essential)
**Sulfenic acid/ Da**	-	103.993
**Crosslinker reactivity**	K-K	K-K
**Fixed modification**	Carbamidomethyl [C]	Carbamidomethyl [C]
**Variable modification**	Oxidation [M]	Oxidation [M]
**Enzyme**	Trypsin	Trypsin
**Max. missed cleavages**	3	R:3 K:3
**Min peptide mass**	500	500
**Max peptide mass**	6000	6000
**Min peptide length**	5	5
**MS1 tolerance (ppm)**	5	5
**MS2 tolerance (ppm)**	20	20
**S/N ratio**	1.5	1.5
**FDR calculation**	inbuilt	inbuilt
**FDR level**	PSM	PSM

Supplementary Table 12; Search settings used for the identification of DSBU- and DSSO- crosslinked peptides.
