# Supplementary material for: A synthetic peptide library for benchmarking crosslinking-mass spectrometry search engines for proteins and protein complexes
Source: Nat Commun. 2020 Feb 6;11:742. doi: 10.1038/s41467-020-14608-2 (PMC7005041; doi:10.1038/s41467-020-14608-2)
Supplement: Supplementary file 2 — Description of Additional Supplementary Files [file 41467_2020_14608_MOESM2_ESM.docx]

**Description of Supplementary Files**

**File Name: Supplementary Data 1**

**Description:** CSV results files for every search output included in the manuscript. A table of contents describes each file in detail.

**File Name: Supplementary Data 2**

**Description:** Configuration settings and fasta files required to perform the database searches with each search engine.
