# Supplementary material for: A synthetic peptide library for benchmarking crosslinking-mass spectrometry search engines for proteins and protein complexes
Source: Nat Commun. 2020 Feb 6;11:742. doi: 10.1038/s41467-020-14608-2 (PMC7005041; doi:10.1038/s41467-020-14608-2)
Supplement: Supplementary file 3 — Supplementary Data 1 [file 41467_2020_14608_MOESM3_ESM.zip › CSVfiles/CSV_ToC.docx]

| File name | Description |
| --- | --- |
| Kojak_peptideprophet_R1 | DSS-crosslinked peptides analysed by Kojak, results validated with Peptide Prophet, unfiltered, Repeat 1 |
| Kojak_peptideprophet_R2 | DSS-crosslinked peptides analysed by Kojak, results validated with Peptide Prophet, unfiltered, Repeat 2 |
| Kojak_peptideprophet_R3 | DSS-crosslinked peptides analysed by Kojak, results validated with Peptide Prophet, unfiltered, Repeat 3 |
| Kojak_peptideprophet_R1_crapome | DSS-crosslinked peptides analysed by Kojak with Crapome database, validated with PeptideProphet, unfiltered, Repeat 1 |
| Kojak_peptideprophet_R2_crapome | DSS-crosslinked peptides analysed by Kojak with Crapome database, validated with PeptideProphet, unfiltered, Repeat 2 |
| Kojak_peptideprophet_R3_crapome | DSS-crosslinked peptides analysed by Kojak with Crapome database, validated with PeptideProphet, unfiltered, Repeat 3 |
| Kojak_percolator_R1_allCSM | Kojak results validated with Percolator on the CSM level, unfiltered, Repeat 1 |
| Kojak_percolator_R2_allCSM | DSS-crosslinked peptides analysed by Kojak, results validated with Percolator on the CSM level, unfiltered, Repeat 2 |
| Kojak_percolator_R3_allCSM | DSS-crosslinked peptides analysed by Kojak, results validated with Percolator on the CSM level, unfiltered, Repeat 3 |
| kojak_percolator_R1_uniqueCSM | DSS-crosslinked peptides analysed by Kojak, results validated with Percolator on the unique-CSM level, unfiltered, Repeat 1 |
| kojak_percolator_R2_uniqueCSM | DSS-crosslinked peptides analysed by Kojak, results validated with Percolator on the unique-CSM level, unfiltered, Repeat 2 |
| kojak_percolator_R3_uniqueCSM | DSS-crosslinked peptides analysed by Kojak, results validated with Percolator on the unique-CSM level, unfiltered, Repeat 3 |
| kojak_percolator_R1_uniqueCSM_crapome | DSS-crosslinked peptides analysed by Kojak with Crapome database, results validated with Percolator on the unique-CSM level, unfiltered, repeat 1 |
| kojak_percolator_R2_uniqueCSM_crapome | DSS-crosslinked peptides analysed by Kojak with Crapome database, validated with Percolator on the unique-CSM level, unfiltered, repeat 2 |
| kojak_percolator_R3_uniqueCSM_crapome | DSS-crosslinked peptides analysed by Kojak with Crapome database, validated with Percolator on the unique-CSM level, unfiltered, repeat 3 |
| pLink_R1_1perFDR | DSS-crosslinked peptides analysed by pLink, 1% FDR, Repeat 1 |
| pLink_R2_1perFDR | DSS-crosslinked peptides analysed by pLink, 1% FDR, Repeat 2 |
| pLink_R3_1perFDR | DSS-crosslinked peptides analysed by pLink, 1% FDR, Repeat 3 |
| pLink_R1_5perFDR | DSS-crosslinked peptides analysed by pLink, 5% FDR, Repeat 1 |
| pLink_R2_5perFDR | DSS-crosslinked peptides analysed by pLink, 5% FDR, Repeat 2 |
| pLink_R3_5perFDR | DSS-crosslinked peptides analysed by pLink, 5% FDR, Repeat 3 |
| pLink_R1_5perFDR_crapome | DSS-crosslinked peptides analysed by pLink with Crapome database, 5% FDR, Repeat 1 |
| pLink_R2_5perFDR_crapome | DSS-crosslinked peptides analysed by pLink with Crapome database, 5% FDR, Repeat 2 |
| pLink_R3_5perFDR_crapome | DSS-crosslinked peptides analysed by pLink with Crapome database, 5% FDR, Repeat 3 |
| R1_10proteins_XiVersion1.6.751 | DSS-crosslinked peptides analysed by Xi with 10 extra proteins in database, unfiltered, Repeat 1 |
| R2_10proteins_XiVersion1.6.751 | DSS-crosslinked peptides analysed by Xi with 10 extra proteins in database, unfiltered, Repeat 2 |
| R3_10proteins_XiVersion1.6.751 | DSS-crosslinked peptides analysed by Xi with 10 extra proteins in database, unfiltered, Repeat 3 |
| R1_1perFDR_PSMlevel_xiFDR1.1.27 | DSS-crosslinked peptides analysed by Xi, 1% FDR at PSM level, Repeat 1 |
| R2_1perFDR_PSMlevel_xiFDR1.1.27 | DSS-crosslinked peptides analysed by Xi, 1% FDR at PSM level, Repeat 2 |
| R3_1perFDR_PSMlevel_xiFDR1.1.27 | DSS-crosslinked peptides analysed by Xi, 1% FDR at PSM level, Repeat 3 |
| R1_5perFDR_PSMlevel_xiFDR1.1.27 | DSS-crosslinked peptides analysed by Xi, 5% FDR at PSM level, Repeat 1 |
| R2_5perFDR_PSMlevel_xiFDR1.1.27 | DSS-crosslinked peptides analysed by Xi, 5% FDR at PSM level, Repeat 2 |
| R3_5perFDR_PSMlevel_xiFDR1.1.27 | DSS-crosslinked peptides analysed by Xi, 5% FDR at PSM level, Repeat 3 |
| R1_5perFDR_uniquePSMlevel_xiFDR1.1.27 | DSS-crosslinked peptides analysed by Xi, 5% FDR at unique PSM level, Repeat 1 |
| R2_5perFDR_uniquePSMlevel_xiFDR1.1.27 | DSS-crosslinked peptides analysed by Xi, 5% FDR at unique PSM level, Repeat 2 |
| R3_5perFDR_uniquePSMlevel_xiFDR1.1.27 | DSS-crosslinked peptides analysed by Xi, 5% FDR at unique PSM level, Repeat 3 |
| R1_5perFDR_peppairlevel_xiFDR1.1.27 | DSS-crosslinked peptides analysed by Xi, 5% FDR at peptide pair level, Repeat 1 |
| R2_5perFDR_peppairlevel_xiFDR1.1.27 | DSS-crosslinked peptides analysed by Xi, 5% FDR at peptide pair level, Repeat 2 |
| R3_5perFDR_peppairlevel_xiFDR1.1.27 | DSS-crosslinked peptides analysed by Xi, 5% FDR at peptide pair level, Repeat 3 |
| R1_crapome_XiVersion1.6.751 | DSS-crosslinked peptides analysed by Xi with crapome database, unfiltered, Repeat 1 |
| R2_crapome_XiVersion1.6.751 | DSS-crosslinked peptides analysed by Xi with crapome database, unfiltered, Repeat 2 |
| R3_crapome_XiVersion1.6.751 | DSS-crosslinked peptides analysed by Xi with crapome database, unfiltered, Repeat 3 |
| R1_crapome_5perFDR_PSMlevel_xiFDR1.1.27 | DSS-crosslinked peptides analysed by Xi with crapome database, 5% FDR at PSM level, repeat 1 |
| R2_crapome_5perFDR_PSMlevel_xiFDR1.1.27 | DSS-crosslinked peptides analysed by Xi with crapome database, 5% FDR at PSM level, repeat 2 |
| R3_crapome_5perFDR_PSMlevel_xiFDR1.1.27 | DSS-crosslinked peptides analysed by Xi with crapome database, 5% FDR at PSM level, repeat 3 |
| R1_crapome_5perFDR_uniquePSMlevel_xiFDR1.1.27 | DSS-crosslinked peptides analysed by Xi with crapome database, 5% FDR at unique PSM level, repeat 1 |
| R2_crapome_5perFDR_uniquePSMlevel_xiFDR1.1.27 | DSS-crosslinked peptides analysed by Xi with crapome database, 5% FDR at unique PSM level, repeat 2 |
| R3_crapome_5perFDR_uniquePSMlevel_xiFDR1.1.27 | DSS-crosslinked peptides analysed by Xi with crapome database, 5% FDR at unique PSM level, repeat 3 |
| R1_crapome_5perFDR_peppairlevel_xiFDR1.1.27 | DSS-crosslinked peptides analysed by Xi with crapome database, 5% FDR at peptide pair level, repeat 1 |
| R2_crapome_5perFDR_peppairlevel_xiFDR1.1.27 | DSS-crosslinked peptides analysed by Xi with crapome database, 5% FDR at peptide pair level, repeat 2 |
| R3_crapome_5perFDR_peppairlevel_xiFDR1.1.27 | DSS-crosslinked peptides analysed by Xi with crapome database, 5% FDR at peptide pair level, repeat 3 |
| Stavrox_R1_1perFDR | DSS-crosslinked peptides analysed by StavroX, 1% FDR, repeat 1 |
| Stavrox_R2_1perFDR | DSS-crosslinked peptides analysed by StavroX, 1% FDR, repeat 2 |
| Stavrox_R3_1perFDR | DSS-crosslinked peptides analysed by StavroX, 1% FDR, repeat 3 |
| Stavrox_R1_5perFDR | DSS-crosslinked peptides analysed by StavroX, 5% FDR, repeat 1 |
| Stavrox_R2_5perFDR | DSS-crosslinked peptides analysed by StavroX, 5% FDR, repeat 2 |
| Stavrox_R3_5perFDR | DSS-crosslinked peptides analysed by StavroX, 5% FDR, repeat 3 |
| Stavrox_R1_5perFDR_reversedecoy | DSS-crosslinked peptides analysed by StavroX with reverse decoy, 5% FDR, repeat 1 |
| Stavrox_R2_5perFDR_reversedecoy | DSS-crosslinked peptides analysed by StavroX with reverse decoy, 5% FDR, repeat 2 |
| Stavrox_R3_5perFDR_reversedecoy | DSS-crosslinked peptides analysed by StavroX with reverse decoy, 5% FDR, repeat 3 |
| MeroX_DSBU_rise | DSBU-crosslinked peptides analysed by MeroX in Rise mode, 5% FDR |
| Merox_DSBU_riseup | DSBU-crosslinked peptides analysed by MeroX in RiseUP mode |
| Merox_DSSO_RISE | DSSO-crosslinked peptides analysed by MeroX in Rise mode |
| Merox_DSSO_riseup | DSSO-crosslinked peptides analysed by MeroX in RiseUP mode |
| xlinkx_QExHFX_DSSO_5perFDR_crapome_CSMs | DSSO-crosslinked peptides measured on Q-exactive, analysed with xlinkx, 5% FDR |
| xlinkx_QExHFX_DSSO_1perFDR_crapome_CSMs | DSSO-crosslinked peptides measured on Q-exactive, analysed with xlinkx, 1% FDR |
| xlinkx_QExHFX_DSSO_cutoff-45-4_CSMs | DSSO-crosslinked peptides measured on Q-exactive, analysed with xlinkx, minimum xlinkx score 45, minimum score difference 4 |
| xlinkx_QExHFX_DSBU_5perFDR_crapome_CSMs | DSBU-crosslinked peptides measured on Q-exactive, analysed with xlinkx, 5% FDR |
| xlinkx_QExHFX_DSBU_1perFDR_crapome_CSMs | DSBU-crosslinked peptides measured on Q-exactive, analysed with xlinkx, 1% FDR |
| xlinkx_Lumos_DSSO_stHCD-MS2_cutoff-45-4_CSMs | DSSO-crosslinked peptides measured on Lumos with stepped-HCD, analysed with xlinkx, minimum xlinkx score 45, minimum score difference 4 |
| xlinkx_Lumos_DSSO_stHCD-MS2_cutoff-20_CSMs | DSSO-crosslinked peptides measured on Lumos with stepped-HCD, analysed with xlinkx, minimum xlinkx score 20, minimum score difference 4 |
| xlinkx_Lumos_DSSO_MS3-EThcD_cutoff-45-4_CSMs | DSSO-crosslinked peptides measured on Lumos with MS3-EThcD, analysed with xlinkx, minimum xlinkx score 45, minimum score difference 4 |
| xlinkx_Lumos_DSSO_MS3_cutoff-45-4_CSMs | DSSO-crosslinked peptides measured on Lumos with MS3, analysed with xlinkx, minimum xlinkx score 45, minimum score difference 4 |
| xlinkx_Lumos_DSSO_CID-ETD_cutoff-45-4_CSMs | DSSO-crosslinked peptides measured on Lumos with CID-ETD, analysed with xlinkx, minimum xlinkx score 45, minimum score difference 4 |
