# Supplementary material for: A synthetic peptide library for benchmarking crosslinking-mass spectrometry search engines for proteins and protein complexes
Source: Nat Commun. 2020 Feb 6;11:742. doi: 10.1038/s41467-020-14608-2 (PMC7005041; doi:10.1038/s41467-020-14608-2)
Supplement: Supplementary file 4 — Supplementary Data 2 [file 41467_2020_14608_MOESM4_ESM.zip › Supplementary Data 2/PDsettings.pptx]

## Slide 1
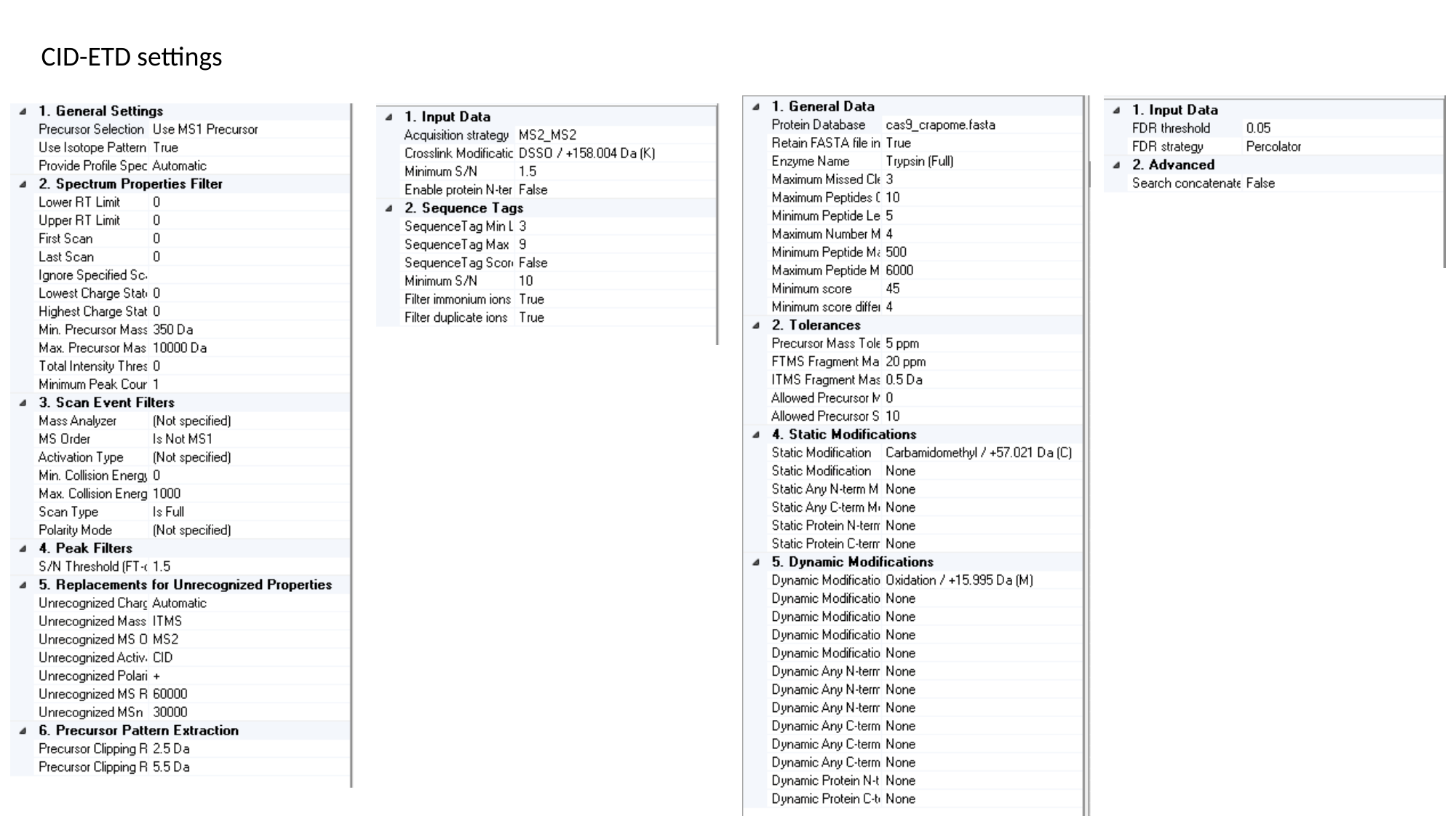

CID-ETD settings

## Slide 2
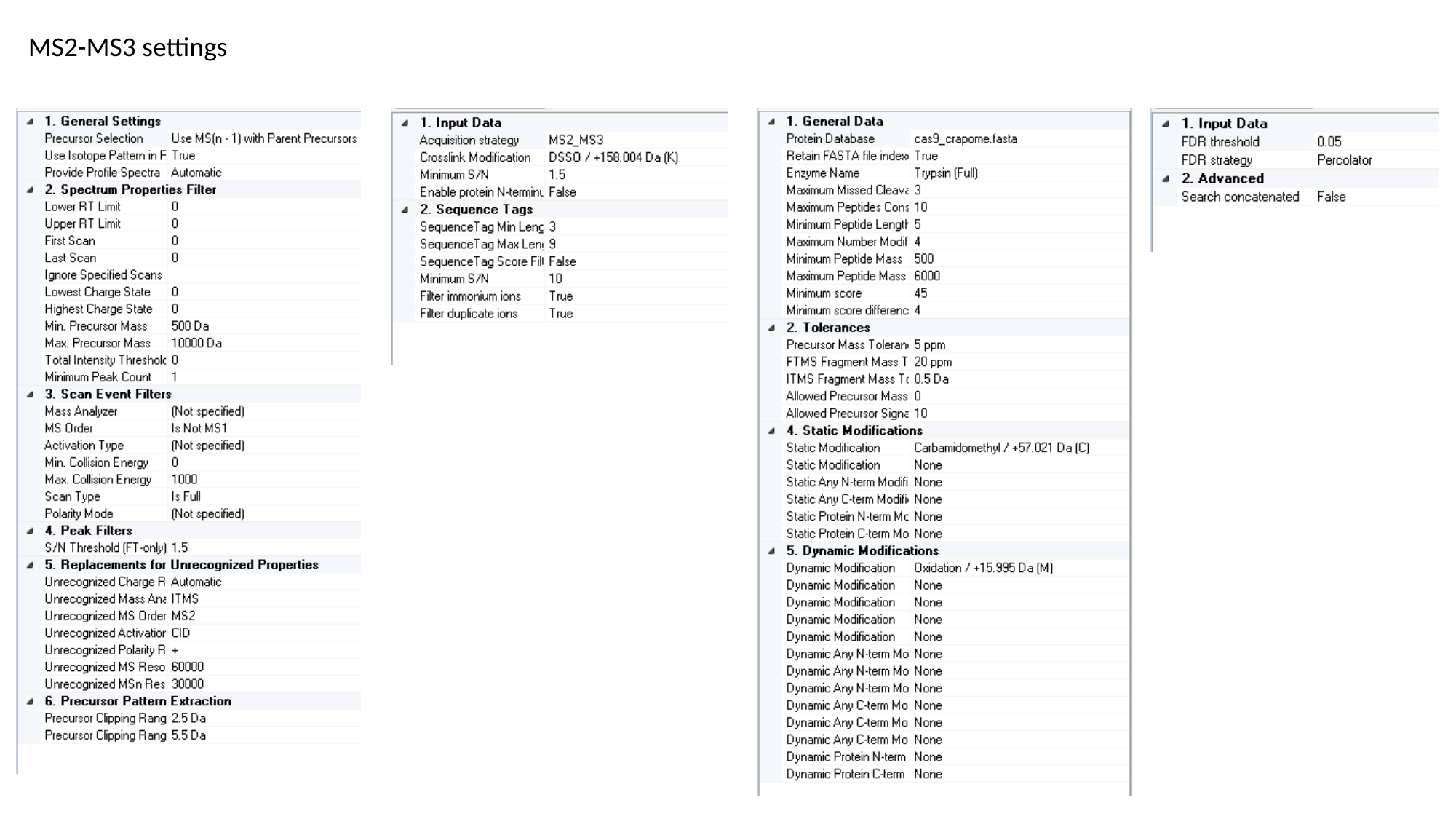

MS2-MS3 settings

## Slide 3
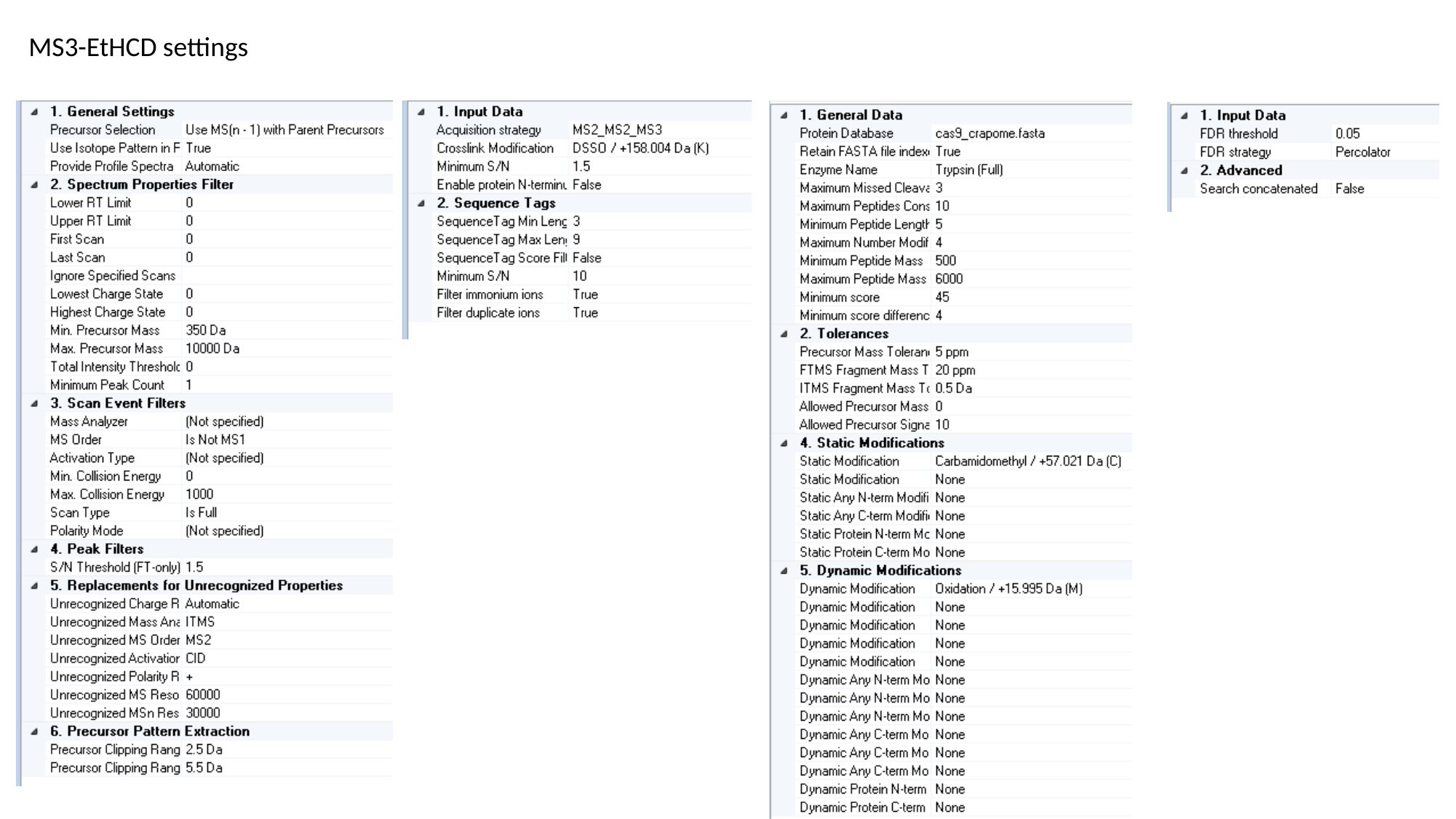

MS3-EtHCD settings

## Slide 4
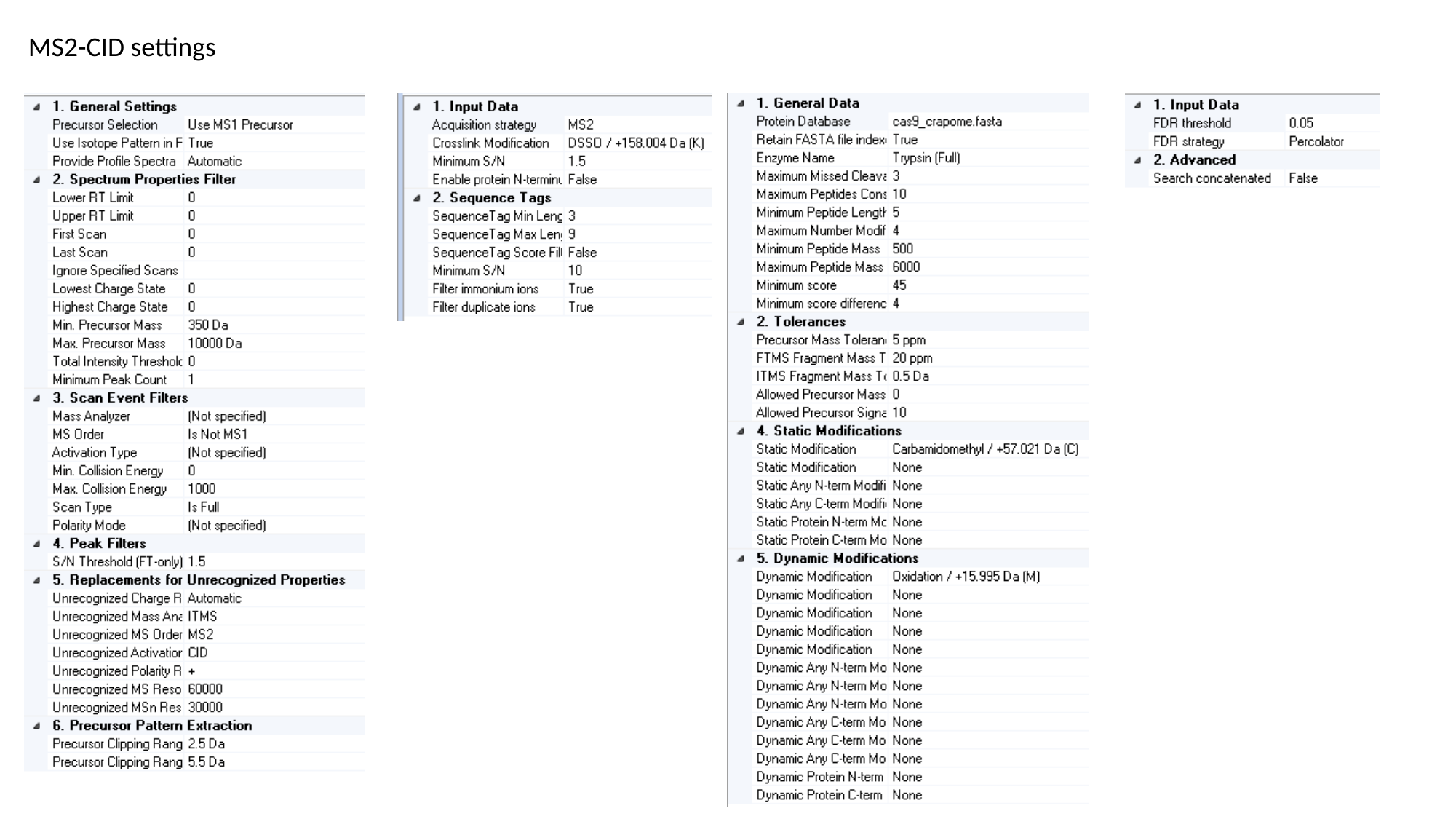

MS2-CID settings

## Slide 5
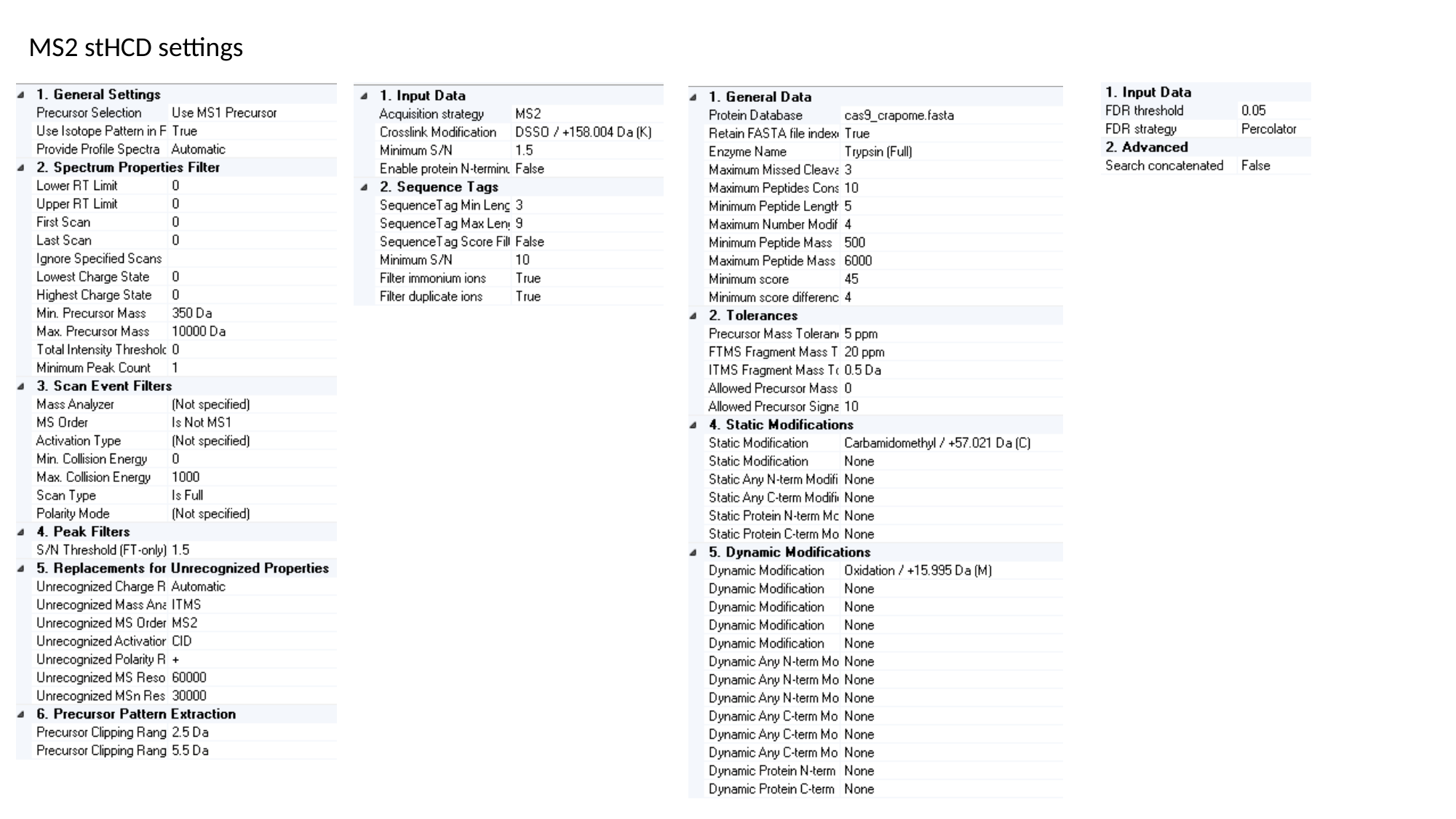

MS2 stHCD settings

## Slide 6
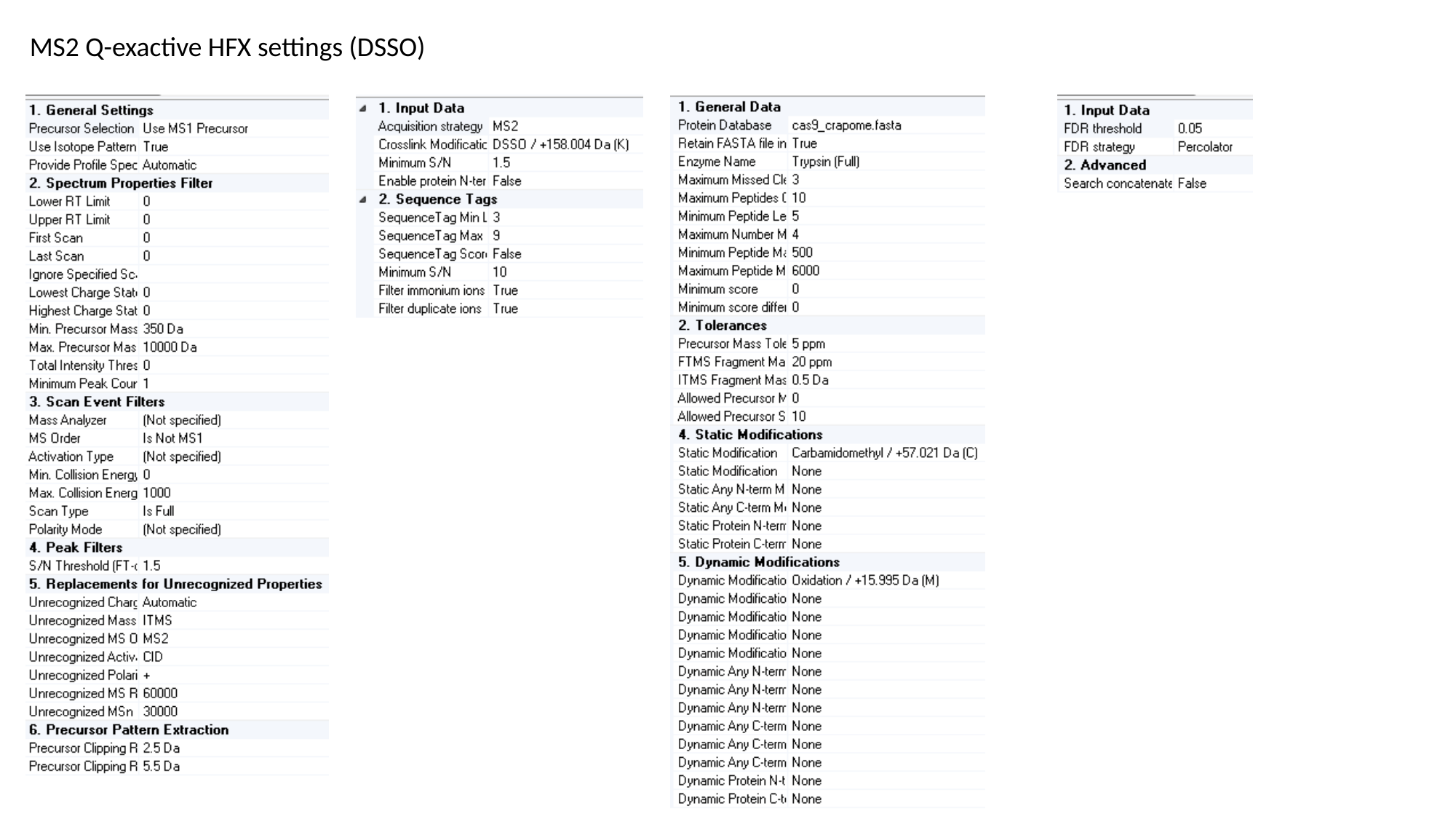

MS2 Q-exactive HFX settings (DSSO)

## Slide 7
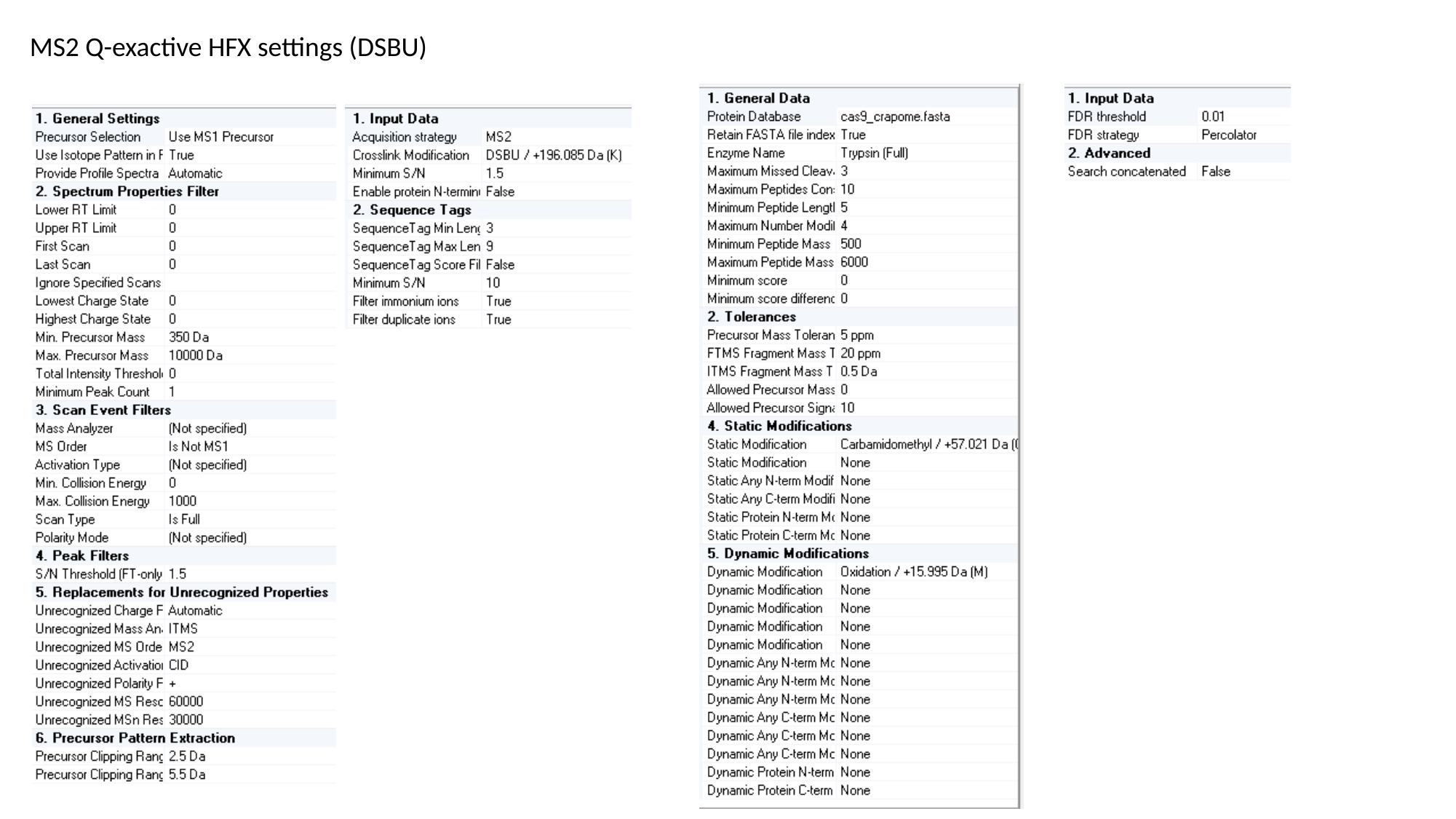

MS2 Q-exactive HFX settings (DSBU)
