# Supplementary figures and images for: A synthetic peptide library for benchmarking crosslinking-mass spectrometry search engines for proteins and protein complexes
Source: Nat Commun. 2020 Feb 6;11:742. doi: 10.1038/s41467-020-14608-2 (PMC7005041; doi:10.1038/s41467-020-14608-2)

## Slide 1
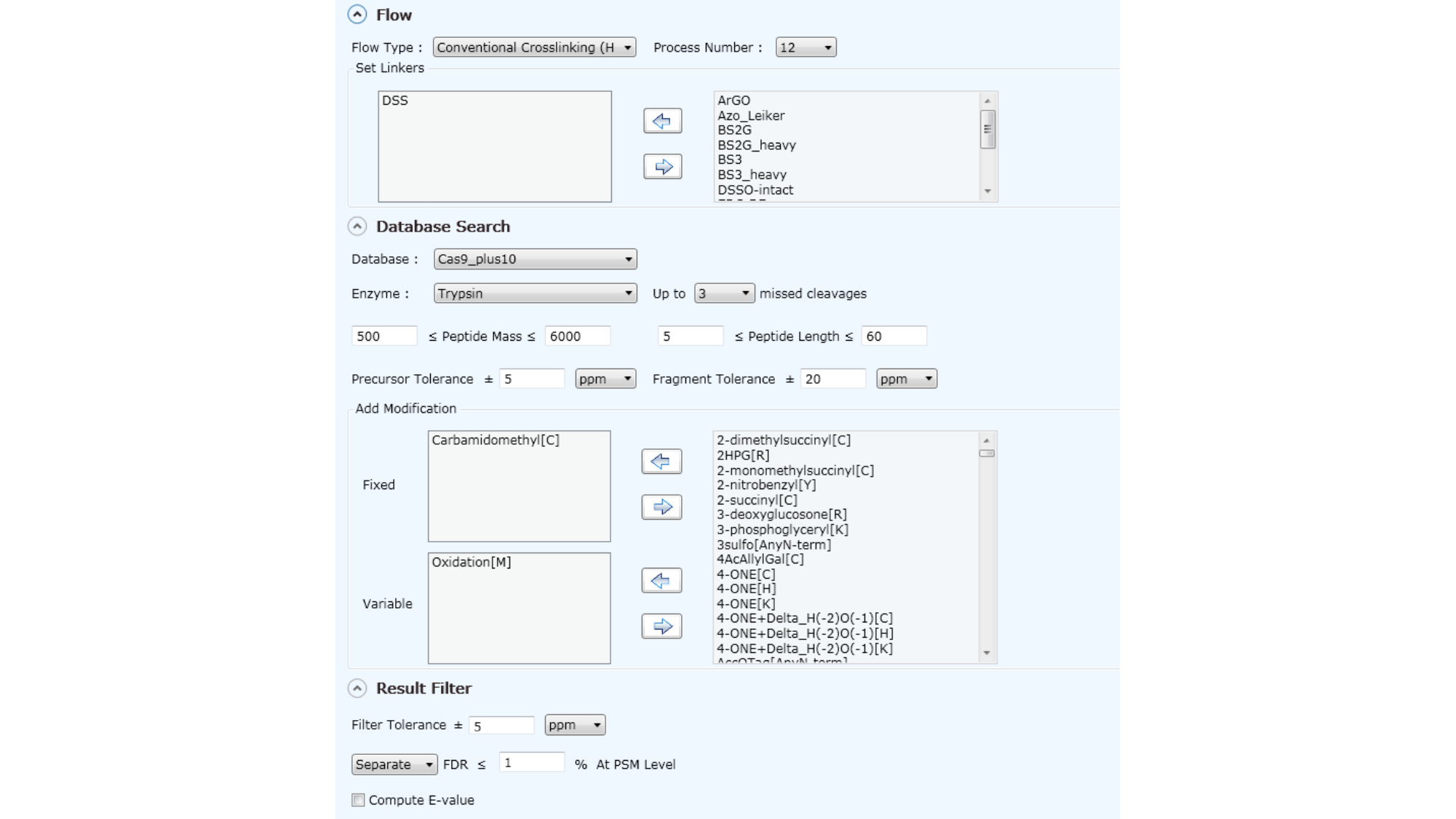

Supplement: Supplementary file 4 — Supplementary Data 2 [file 41467_2020_14608_MOESM4_ESM.zip › Supplementary Data 2/pLinksettings.pptx]
